# Abundance of *Dendroctonus frontalis* and *D*. *mexicanus *(Coleoptera: Scolytinae) along altitudinal transects in Mexico: Implications of climatic change for forest conservation

**DOI:** 10.1371/journal.pone.0288067

**Published:** 2023-07-05

**Authors:** Cuauhtémoc Sáenz-Romero, Víctor Hugo Cambrón-Sandoval, William Hammond, Jorge Méndez-González, Hugo Luna-Soria, Jorge E. Macías-Sámano, Mariela Gómez-Romero, Oscar Trejo-Ramírez, Craig D. Allen, Erika Gómez-Pineda, Ek del-Val

**Affiliations:** 1 Instituto de Investigaciones sobre los Recursos Naturales, Universidad Michoacana de San Nicolás de Hidalgo, Morelia, Michoacán, México; 2 Facultad de Ciencias Naturales, Universidad Autónoma de Querétaro, Querétaro, Querétaro, México; 3 Agronomy Department, University of Florida, Gainesville, Florida, United States of America; 4 Departamento Forestal, Universidad Autónoma Agraria Antonio Narro, Saltillo, Coahuila, México; 5 Forest Health and Semiochemicals Consulting, Coquitlam, British Columbia, Canada; 6 Facultad de Biología, Universidad Michoacana de San Nicolás de Hidalgo, Morelia, Michoacán, México; 7 Cátedras of the Consejo Nacional de Ciencia y Tecnología, Ciudad de México, México; 8 Dirección General de Gestión Forestal y de Suelos, Secretaría de Medio Ambiente y Recursos Naturales, Ciudad de México, México; 9 Department of Geography and Environmental Studies, University of New Mexico, Albuquerque, New Mexico, United States of America; 10 Instituto de Investigaciones Agropecuarias y Forestales, Universidad Michoacana de San Nicolás de Hidalgo, Morelia, Michoacán, México; 11 Centro de Investigaciones en Geografía Ambiental, Universidad Nacional Autónoma de México, Morelia, Michoacán, México; 12 Instituto de Investigaciones en Ecosistemas y Sustentabilidad, Universidad Nacional Autónoma de México, Morelia, Michoacán, México; Veracruzana University: Universidad Veracruzana, MEXICO

## Abstract

Bark beetle infestations have historically been primary drivers of stand thinning in Mexican pine forests. However, bark beetle impacts have become increasingly extensive and intense, apparently associated with climate change. Our objective was to describe the possible association between abundance of bark beetle flying populations and the occurrence of given value intervals of temperature, precipitation and their balance, in order to have a better comprehension of the climatic space that might trigger larger insect abundances, an issue relevant in the context of the ongoing climatic change. Here, we monitored the abundance of two of the most important bark beetle species in Mexico, *Dendroctonus frontalis* and *D*. *mexicanus*. We sampled 147 sites using pheromone-baited funnel traps along 24 altitudinal transects in 11 Mexican states, from northwestern Chihuahua to southeastern Chiapas, from 2015 to 2017. Through mixed model analysis, we found that the optimum Mean Annual Temperatures were 17°C–20°C for *D*. *frontalis* in low-elevation pine-oak forest, while *D*. *mexicanus* had two optimal intervals: 11–13°C and 15–18°C. Higher atmospheric Vapor Pressure Deficit (≥ 1.0) was correlated with higher *D*. *frontalis* abundances, indicating that warming-amplified drought stress intensifies trees’ vulnerability to beetle attack. As temperatures and drought stress increase further with projected future climatic changes, it is likely that these *Dendroctonus* species will increase tree damage at higher elevations. Pine forests in Mexico are an important source of livelihood for communities inhabiting those areas, so providing tools to tackle obstacles to forest growth and health posed by changing climate is imperative.

## 1. Introduction

Mexico is considered the center of origin and diversification for *Pinus* tree species [1], and there is evidence of a parallel, co-evolved, regional diversification of the bark beetle genus *Dendroctonus* (Coleoptera: Curculionidae: Scolytinae) [2,3]. Multiple species of *Dendroctonus* bark beetles are aggressive tree-killing pests of pine forests in North and Central America [4–6]. In Mexico the genus *Dendroctonus* contains abundant and diverse bark beetle species, with 13 beetle species that colonize 24 of Mexico’s 47 pine species [3,7]. An assessment of pine vulnerability to bark beetles in Mexico’s forests shows that high pine diversity is a major determining risk factor at the regional level; the most affected regions are the Transverse Volcanic Belt, followed by the Sierra Madre Occidental and Sierra Madre del Sur [5]. At the local level, anthropic and natural disturbance are the key risk factors behind vulnerability [8].

Native bark beetles contribute to the maintenance of ecologically healthy and vigorous forests by removing old, sick, and stressed individuals [9,10]. In this role, bark beetles represent a natural and constant disturbance force in the forest [11,12]. Stand factors such as age structure, species distribution, basal area, site index, etc., influence the risk of large bark beetle populations by acting on host tree resource availability and even on production and mobility of aggregation pheromone plumes that concentrate beetle mass-attack colonization of vulnerable trees [9,11,13]. Successful colonization and reproduction of bark beetles in living trees requires the release of enough aggregation pheromone to ensure that sufficient conspecifics are attracted to the host tree to overwhelm its defenses [14]. However, many other intrinsic biological processes of the beetles are involved. For example, the particular timing of beetle flight and the distance they can cover are related to lipid reserves on emergence and lipid metabolism in flight [15,16].

An increasing number of studies have linked climate change to an increase of bark beetle outbreaks, especially heat and drought, which are the climate factors most clearly causally linked to host tree stress [11,17–22]. Host tree stress makes them more vulnerable to insect pests, particularly bark beetles [13,23–26]. There is evidence that climate change is now altering the life cycles and distributions of multiple bark beetle species around the world [27–29]. For example, the populations of *Dendroctonus ponderosae* Hopkins [30,31] and *D*. *frontalis* Zimmerman [32,33] have increased, since milder winter temperatures are killing fewer individuals. Also, some species are experiencing an increase in the number of generations per year, such as *Ips typographus* in Europe [34] and *D*. *rufipennis* in North America [35].

For Mexico studies are scarcer, but bark beetle outbreaks appear to have worsened since 1970, and especially strongly since about 2010 [21,36]. For example, between 2011 and 2013, there were unusually large bark beetle outbreaks of *D*. *mexicanus* Hopkins, *D*. *frontalis*, and *Ips lecontei* Swaine (D. Cibrián-Tovar, Universidad Autónoma Chapingo, México, pers. com.). During 2010–2012 the state of Durango lost 25% of its forest production and between 2012 and 2013, 43,859 hectares of forest in Chihuahua were damaged by bark beetles [37]. Similar situations have occurred in Honduras and Guatemala with *D*. *frontalis* [38]. After a report of increased bark beetle incidence in Michoacán in west-central Mexico, Rubin-Aguirre *et al*. [39] studied Scolytinae communities and found greater beetle prevalence at lower elevations, correlated with higher maximum temperatures.

Despite correlations with temperature, the role of climate in bark beetle outbreaks is not yet fully understood [40]. Turchin *et al*. [41] analyzed 30 years of *D*. *frontalis* population data in East Texas, revealing that these bark beetles respond to a delayed density-dependent process. However, Friedenberg *et al*. [33], working with models that combine both density-dependent population regulation and climate variables over a large area across 48 years, found that population growth declined with the number of days exceeding 32°C and that density dependence also decreased with the number of extremely cold (- 4.32°C) days. These results suggest that although increasingly warm temperatures (due to climatic change) could favor larger bark beetle populations, there is an upper limit of favorable temperatures, and importantly, extreme events (e.g., heatwaves) may have a negative effect [42].

Monitoring bark beetle populations while they are flying has been done successfully using traps baited with their own aggregation pheromones [43]. These pheromone traps are routinely used in government programs in Mexico and Central America [44]. Based on that previous experience, in 2014 the Mexican National Forest Commission (CONAFOR, for its acronym in Spanish), along with the Mexican Council of Science and Technology (CONACyT), promoted and funded studies on the abundance of the three most important pine bark-beetle species in the country—*D*. *frontalis*, *D*. *mexicanus* and *D*. *adjunctus* Blandford [3,45]—using pheromone-baited traps along altitudinal gradients [46]. Initial findings of this project showed that the most critical months for the abundance of flying *Dendroctonus* spp. are from March to May, corresponding with the warm dry season [47–50]. Altitudinal patterns of several *Dendroctonus* species are also known from entomological collections across the country [8,51], where *D*. *frontalis* is primarily distributed at lower elevations (below 2,000 m a.s.l.) than *D*. *mexicanus*. This finding is consistent with studies using pheromone-baited traps at different elevations in Querétaro [46,52].

Although bark beetles outbreaks are an increasing management problem in Mexican temperate forests, there are no previous nation-wide studies that simultaneously coordinate trapping of flying bark beetle populations and measure climate variables right at the trapping sites. In order to be able to link the abundance of these populations at a given climatic intervals, a large scale analysis of a possible association between the two was conducted for this work. We aimed to contribute to fill the knowledge gaps, especially related to the association between bark beetle abundance and climate, a critical information under the context of climatic change. We explored the limits of the climatic space occupied by each bark beetle species by conducting a trapping along altitudinal gradients. Given the strong association between temperatures and elevation on the complex Mexican mountain ranges (see Fig 2 on [53]), we developed a sampling strategy that provides an array of a diverse occurrence of temperatures and precipitations on each of the studied locations. This sampling approach along elevational gradients, could be applied outside México, like in Guatemala, Belize, Honduras and Nicaragua, that not only share the same bark beetle problems, but also share several keystone pine species with México at low and high elevations, like *Pinus oocarpa* and *P*. *hartwegii*, respectively, as well as *Dendroctonus* species like *Dendroctonus frontalis*, *D*. *adjunctus*, *D*. *mesoamericanus*, *D*. *valens* and *D*. *aproximatus*, among others [7,54,55].

Thus, we present a nation-wide analysis of the CONAFOR-CONACyT project on the abundances of *D*. *frontalis* and *D*. *mexicanus* across elevational gradients and the relationship between beetle abundance and local measurements of air temperature and relative humidity and estimates of precipitation and evapotranspiration. **The** present work aims to determine the climatic envelopes occupied by each of these bark beetle species at the broadest scale across Mexico, rather than building site-based predictive models that would require more detailed parameterization of site-specific conditions (e.g., stand characteristics, insect populations). After determining these national-scale climatic envelopes, we discuss the possible consequences for bark beetle abundance in Mexico under ongoing and projected future climate change conditions.

## 2. Materials and methods

### 2.1 Study sites

**Fig 1:**
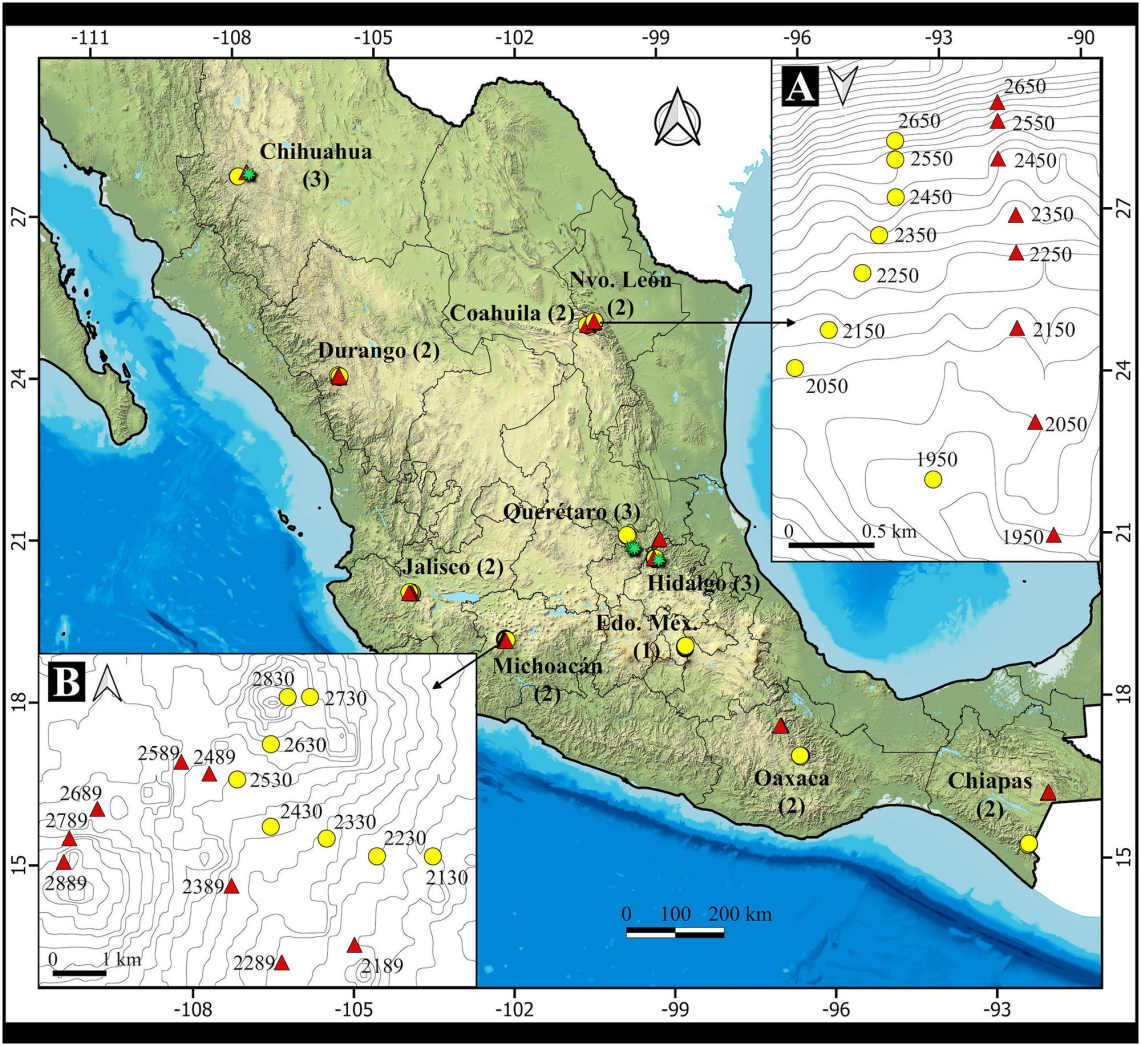
Geographic location of the altitudinal transects (red symbols) along which traps were installed. In parenthesis the number of transects per State. Upper right and lower left panels zoom on transects on Nuevo León and Michoacán states, respectively; elevation of traps (m asl) is indicated. More details of transects on S1 to S11 Tables in S1 File. The shapefile was obtained from the as TIFF with a 15m resolution. Source: INEGI, Continuo de Elevaciones Mexicano 3.0 (CEM 3.0), Ver: 202303311214 (https://www.inegi.org.mx/app/geo2/elevacionesmex/). The figure and contour lines were drawn by E.Gómez-Pineda in QGIS 3.10 (https://qgis.org/es/site/). This figure is to be published under the CC-BY 4.0 license.

The study was conducted in the temperate pine and pine-oak forests of 11 Mexican States distributed in the northern, central and southern parts of the country (Fig 1). The aim was to sample populations of *Dendroctonus frontalis* and *D*. *mexicanus* species along altitudinal forest gradients. The range of altitudes covered by the one to three transects per state are summarized in Table 1, and more details on each transect are provided in S1 to S11 Tables in S1 File.

**Table 1:** Summary of Mexican Regions, States, altitudinal range, vegetation and dominant tree species covered by the altitudinal transects. States are sorted from north to south and west to east. More details of individual transects are in S1 to S11 Tables in S1 File.

State	Locality	Altitudinal range (m a.s.l)	Vegetation	Dominant pine species
Northern Mexico				
Chihuahua	Bocoyna	2362–2648	Pine-oak	*P*. *durangensis*
Durango	San Dimas	2300–2600	Pine-oak	*P*. *leiophylla*
Coahuila	Arteaga	2650–3350	Conifers	*P*. *hartwegii*
Nuevo León	Santiago	1950–2650	Pine-oak	*P*. *teocote*
Central Mexico				
Jalisco	Tecolotlán	1999–2306	Pine-oak	*P*. *teocote*
Michoacán	Nuevo San Juan Parangaricutiro	2130–2889	Pine-oak	*P*. *pseudostrobus*
México	Tlalmanalco	3152–3611	Conifers	*P*. *hartwegii*
Querétaro	Arroyo Seco, Landa de Matamoros & Pinal de Amoles	1586–3058	Pine-oak	*P*. *greggi*
Hidalgo	Zimapán/Jacala	1342–2611	Pine-oak	*P*. *pseudostrubus*
Southern México				
Oaxaca	Sta. Catarina Lachatao & St. Reyes Pápalo	2113–2899	Pine-oak	*P*. *pseudostrobus*
Chiapas	Las Margaritas & Motozintla	1437–2150	Pine-oak	*P*. *oocarpa*

The transects are located in all the four Mexican geographic regions where the occurrence of *D*. *mexicanus* was primary studied by Pérez-Miranda et al., [56]: the Sierra Madre Occidental, Sierra Madre Oriental, Transverse Volcanic Belt and Sierra del Sur.

The transects cover the two pine species that conform the extremes of the pine and pine oak forest in México: The transects at Sierra de Arteaga, Coahuila state, includes pine forests dominated by *Pinus hartwegii*, a high altitude pine that conforms the timberline at México, that reach 4,000 m of altitude and dominate several Natural Protected Areas on the highest mountains of México [57]. On the other elevational extreme, transects at Chiapas state covers a pine forest dominated by *P*. *oocarpa*, that neighbor with tropical dry forest, and is also the most important resin producer in México [58]. *Pinus leiophylla*, a pine widely used also as resin producer [59], is also covered on transects at Durango. Transects at forest dominated by *P*. *durangensis* and *P*. *pseudostrobus*, at Chihuahua and Michoacán states, respectively, covers two economically very important pine species for commercial timber exploitation: *P*. *durangensis* for its large distribution at Sierra Madre Occidental, and *P*. *pseudostrobus* for its also wide distribution at Transverse Volcanic Belt at central México and on Sierra Madre del Sur (the last covered by transects at Oaxaca state), a species characterized by a fast growing rate and excellent stem form [60]. Forest dominated by *P*. *greggi*, a species widely used in México on degraded pine forest ecological restoration projects [61] is covered by transects on Querétaro state. Forest dominated by *P*. *teocote*, that grows frequently on sites relatively marginal in moisture [62], is covered by transects at Nuevo León and Jalisco.

The enlisted pine species covered by the transects, were dominant in the stands where the traps were placed (Table 1). However, there are more pine species present on the sampling sites. It is important to highlight that Mexico is a megadiverse country, center of pine speciation, with about 47 species of the genus *Pinus* which represent about 42% of the World total number of pine species [1,63,64]. Historically, México has been a country donor of pine species germoplasm as source for pine plantations as exotics, like *P*. *patula* in South Africa, to say just one example [65]. Because of that, the Mexican pine forests can be seen as a Worldwide natural reservoir of germplasm for contemporary and potential use in ecological restoration and commercial forest plantations, an asset eventually endangered by the ongoing climatic change [22].

### 2.2 Sampling

The elevation gradient included in the sampling sites covers the main distribution ranges of each bark beetle species: 1500–2000 m a.s.l. for *D*. *frontalis* and 2000–2500 m a.s.l. for *D*. *mexicanus* [8,51]. A total of 147 sampling sites were established on 24 altitudinal gradients. The lowest and highest elevation at which traps were placed was 1342 and 3611 m a.s.l., respectively. Each altitudinal transect had between 4 and 10 traps, placed sequentially every 100 m of altitudinal change (see S01 to S11 Tables in S1 File for details). The length of the altitudinal transects also aimed to cover the full natural altitudinal distribution of the most economically and/or ecologically important pine species in each studied region.

For example, in the case of the State of Michoacán, two transects were placed to cover the natural altitudinal distribution of *Pinus pseudostrobus* Lind., the most economically and ecologically important pine species of the Mexican Transvolcanic Belt in that State. On each of those two transects, eight trapping sites were placed, covering an altitudinal range of 700 m of elevational difference. The slope of the transects are illustrated for two states with zoom panels in Fig 1.

At each of the 147 sampling sites, 12-unit Lindgren funnel traps were placed and baited with a mixture of frontalin, racemic *endo*-brevicomin and turpentine from Synergy Semiochemicals Corporation, Canada. Release devices for each semiochemical were as follows: frontalin was dispensed from an Eppendorf tube inside of a plastic sachet (release rate 12 mg / day, chemical purity racemic); *endo*-brevicomin was dispensed from a flexlure (release rate 0.15 mg/ day, chemical purity racemic); and turpentine was dispensed from a single sealed plastic bag (8 × 19 cm) (release rate 3.6 g / day, chemical purity 70:30 mixture of alpha- and beta-pinene). Semiochemicals were replaced every 45 days in the field. Each Lindgren trap had a collecting cup filled with antifreeze solution (PRESTONA AF EX composed mainly of ethylene glycol) to preserve the beetles [66].

Every two weeks traps were visited, antifreeze was replaced, and trapped insects were collected and stored in 70% ethanol. Insect trapping started on March 1^st^, 2015, and ended for most of the states on March 30^th^, 2017, for a collection period of two full years. However, some transects in the following states had to end observations earlier: Michoacán (March 30^th^ 2016, although climate was recorded until March 2017), Chiapas (December 30^th^ 2016), Mexico State (November 15^th^ 2016), and Hidalgo (30^th^ December 2016). Thus the total abundance of trapped insects was estimated separately for each of the two years analyzed, by obtaining the sum of insects trapped by insect species per each trap for each year, where the operative definition of year was as follows. Hereafter, we refer to the “year 2015” as March 1^st^ 2015 to February 28^th^ 2016, and “year 2016” as March 1^st^ 2016 to February 28^th^ 2017. In Michoacán and Chiapas, only “year 2015” was considered. Referring to the “year” as March through February of the following year corresponds more closely to the actual seasons of ecological activity in Mexican pine and pine-oak temperate forests than the calendar year. The warm dry season occurs from March through May, the rainy season from June to October, and the cold dry season from November to February. Seasonal and annual climate estimates were calculated for these periods. Our operative definition of seasons and start and end of year is in general coincidental with the definition of climatic regions of México by Vidal-Zepeda [67], for the corresponding climatic regions of: North, Northeast, Center, Balsas Depression and Oaxaca Valleys and Southeast. In all those regions, in general, March is a month where temperature increases as a transition from colder winter temperatures to the warmer temperatures of Spring and Summer; also, June to October are months that are included in the rainy season of such regions.

All individuals of *D*. *frontalis* and *D*. *mexicanus* were identified and counted to estimate the abundance of bark beetle flight activity as a proxy of the abundance of those insects. Taxonomical identification was done following Armendáriz-Toledano *et al*. [7]. Specimens were collected in accordance with all applicable Mexican regulations, and a sample of the specimens from each locality were deposited at the Colección de Insectos of the Facultad de Biología, Universidad Autónoma de Querétaro. Field experiments were approved by the National Science Research Council (CONACYT) and the Comisión Nacional Forestal (CONAFOR) (Permit number CONACYT-CONAFOR-2014-C01-234547) and by the Dirección General de Vida Silvestre SGPA/DGVS/02594/16. Since we did not worked or interviewed people we did not ask for a informed consent.

### 2.3 *In situ* climate recording

Air temperature and relative humidity were recorded hourly with data loggers (Hobo Microbaq EL-USB-2) over the same periods as the insect trapping. One data logger was placed adjacent to each trap. Daily average, minimum, and maximum temperature were calculated, which were used to calculate the average, minimum, and maximum monthly values for air temperature and relative humidity, using SAS statistical software [68]. Additional derived variables, such as Mean Annual Temperature (MAT), Mean Temperature of the Coldest Month (MTCM) and of the Warmest Month (MTWM) were estimated in order to represent the climate of each site. We also calculated the mean temperatures over the March-May warm dry season because that was the peak period of bark beetle collection in many traps.

### 2.4 Climate variables from other sources

Available soil moisture is a critical environmental factor influencing tree growth and vigor, and therefore affects resistance to herbivory, especially from bark beetles [13,25,69,70]. Also, relative humidity (atmospheric moisture) is known to affect insect performance by accelerating or delaying metamorphosis and growth rate [71]. Our *in situ* data collection did not include measurements of precipitation. We therefore complemented our climatic data with additional information from the TerraClimate website (https://doi.org/10.1038/sdata.2017.191), a globally gridded climate and hydroclimate product that provide (additional than other web sites resources) climatic variables useful to explore the environmental stress induced by hotter droughts, which is a feature characteristic of the ongoing climatic change [22,72]. From that source, we added the following spatially-interpolated climate variables to our analyses of each trap site for each month: vapor pressure deficit (VPD, effectively a measure of atmospheric drought, or the air’s demand for moisture); climatic water deficit (CWD, the difference between precipitation and potential evapotranspiration); soil moisture (SOIL_M, mm of soil extractable water); precipitation (PPT); the Palmer Drought Severity Index (PDSI, a long-term index of soil drought); and the Standardized Precipitation-Evapotranspiration Index (SPEI). From the monthly values per trap, we estimated annual means and means for the warm dry season in March-May. The main climate variables used in this study are described in Table 2.

**Table 2:** Climatic variables used in this study. These climate variables also were estimated for the dry and warm season of March-May. The first three variables were estimated from data collected with dataloggers *in situ*, and the others from TerraClimate (https://doi.org/10.1038/sdata.2017.191).

Code	Definition
MAT	Mean annual temperature (°C)
MTCM	Mean Temperature of the Coldest Month (°C)
MTWM	Mean Temperature of the Warmest Month (°C)
TMAX	Mean annual maximum temperature (average of the monthly maximum temperatures across the year, °C)
TMIN	Mean annual minimum temperature (average of the monthly maximum temperatures across the year, °C)
MAP	Mean Annual Precipitation (mm)
SOIL_M	Mean annual soil moisture (soil extractable water, mm)
PET	Mean annual potential evapotranspiration (mm)
AET	Mean annual actual evapotranspiration (mm)
DEF	Mean annual Climatic Water Deficit (PET-AET) (mm)
VPD	Mean annual Vapor Pressure Deficit (kPa)
PDSI	Mean annual Palmer drought severity index (Relative to the long term trend 1958–2019) (index)
SPEI	Mean Annual Standardized Precipitation-Evapotranspiration Index (index)

In order to explore if the studied years (2015–2016) were somehow atypical or not in the context of a larger time period, we examined the Mean Annual Temperature from the center of each transect for each year from 1958–2021 with data obtained from TerraClimate website.

### 2.5 Statistical analyses

To visualize how the *Dendroctonus* species are distributed across the climate space of our study, we constructed histograms of the sum of insects per species per trap, and then summed those values within each one degree Celsius of Mean Annual Temperature.

Next, we fit a response function curve for each species using abundance of the *Dendroctonus* species per year per trap as the dependent variable, as a function of each climatic variable enlisted on Table 2, either temperatures recorded *in situ* (obtained by averaging first by day and then by month the raw temperature data of the field dataloggers) or climate variables related to moisture obtained from TerraClimate (as described earlier). We used a quadratic mixed model (Proc Mixed of SAS, 2021), following a similar statistical analysis to the one conducted by Leites *et al*. [73,74] and Sáenz-Romero *et al*. [75,76]. The underlying assumption of the quadratic model was that *Dendroctonus* species would have an optimum climate interval where it would be most abundant, with lower abundances at either colder or warmer temperatures than the optimum [77,78].

The model was:

$${\text{Y}}_{ijkl}=\beta_{0}+\beta_{1}{\text{C}}_{i}+\beta_{2}{\text{C}}_{i}{}^{2}+\beta_{3}{\text{S}}_{j}+\beta_{4}\text{T}{\left(\text{S}\right)}_{k(j)}+{\text{e}}_{ijkl}$$ (1)

Where: Y_*ijkl*_ = observation of number of insects trapped of a given species. β _0_ … β _4_ = Regression parameters. C_*i*_ = *i*^th^ climate variable. S_*j*_ = *j*^th^ State. T(S) _*k(j)*_ = *k*^th^ Transect nested in the *j*^th^ State, and e_*ijkl*_ = error term. The climate variable (C_*i*_) was a fixed term, while state and transect nested in state were the random terms. Year of observation (March 2015 to February 2016, March 2016 to February 2017) was considered as a *repetition* and its effect was also included in the error term.

The best model (i.e., the set of climate variables that best fit the response function curve), was chosen based on a combination of three criteria: (a) the lowest value of the Akaike Information Criterion (AIC, [79]), (b) the climatic quadratic term needed to be negative, and (c) at least one of the climatic terms should be statistically significant (P ≤ 0.05).

After a preliminary analysis, we found that for each species there were many traps with zero individuals or very low abundance (< 50 individuals) of a given *Dendroctonus* species, likely because the geographic and altitudinal location of such traps were outside the main distribution area of the given species. Thus, to avoid an undesirable bias of fit on response functions due to excessive weight of the zero or very low abundance values, we decided (based on local expertise about each species) to set a minimum abundance of captured insects per year per trap to be included in the mixed-model analyses as follows: 50 individuals for *D*. *mexicanus* and 500 for *D*. *frontalis*; such criteria was applied when doing the analysis per *Dendroctonus* species.

## 3. Results

### 3.1 Distribution of *Dendroctonus* spp. abundance along the climatic space

**Fig 2:**
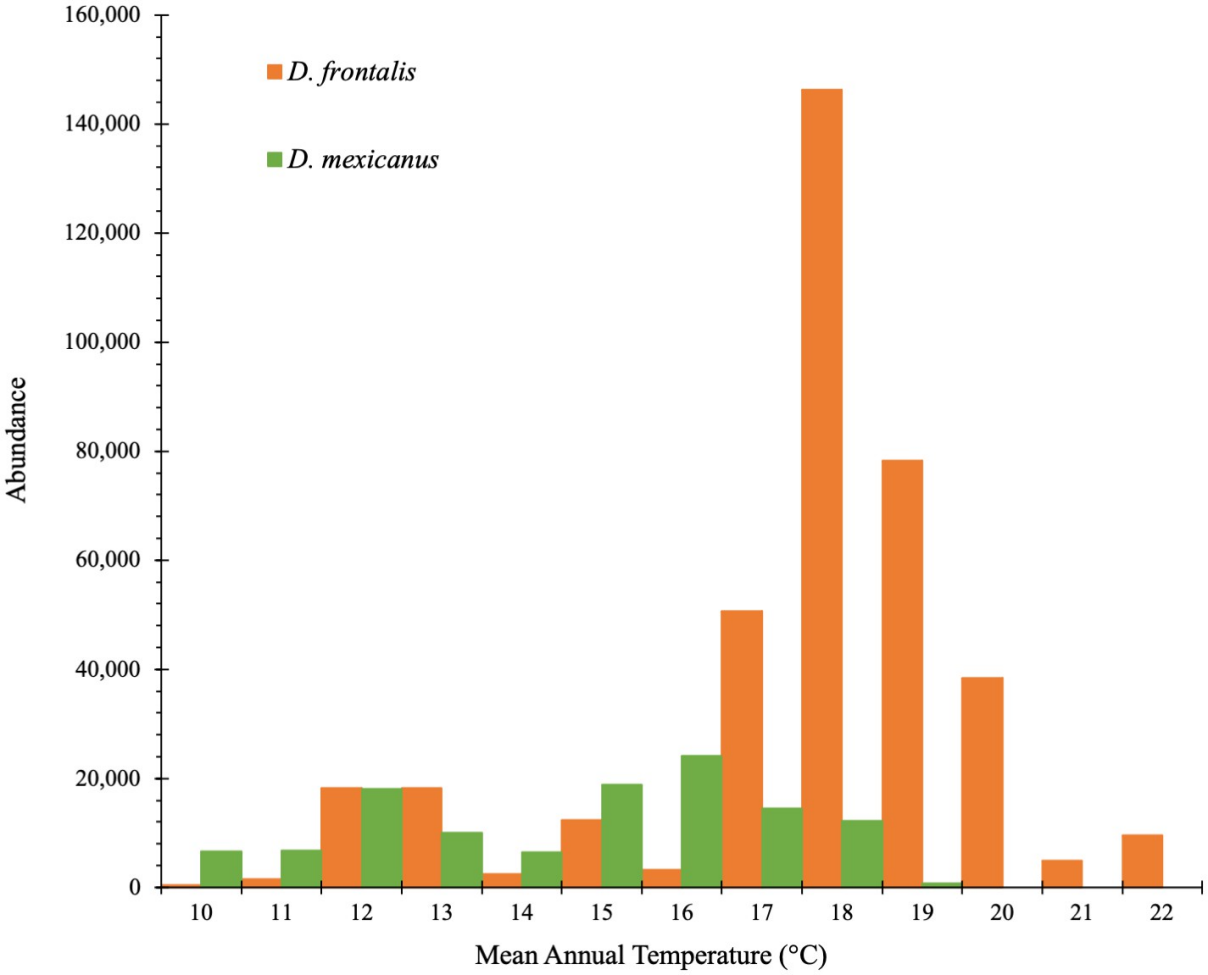
Total abundance of *Dendroctonus* spp. captured by trap and per year from all traps during the entire study and summed by intervals of 1°C of Mean Annual Temperature, for *Dendroctonus frontalis* and *D*. *mexicanus*.

We found the greatest abundance of *D*. *frontalis* between 17 and 20°C MAT, with an optimum at 18°C. The number of individuals trapped between 18.0 and 19.0°C in one year reached 146,388, by far the highest abundance found during our study (Fig 2; for a histogram by species see S1a Fig).

We observed a less clear distribution pattern for *D*. *mexicanus* in relation to temperature. The highest abundances ranged between 11 to 13°C of MAT, with an optimum at 12°C (which mostly corresponds to conifer forests). A second interval of high abundances appeared at warmer sites, between 15°C and 18°C of MAT (corresponding to pine-oak forest), with an optimum at 16°C. Note that the second distribution interval of *D*. *mexicanus* widely overlaps with that of *D*. *frontalis* (Figs 2 and S1b).

### 3.2 Climatic response functions

**Fig 3:**
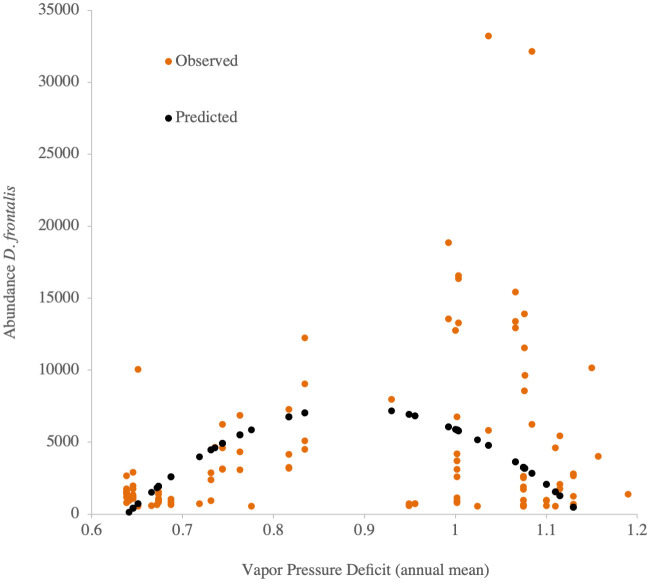
Abundance (total per year per trap) of *Dendroctonus frontalis* graphed by Vapor Pressure Deficit (annual mean, kPa). Predicted data are estimated solely from the fixed terms of a quadratic response function in a mixed model, as in Table 2.

When fitting the mixed model of the bark beetle abundance per species captured per year per trap against the climatic variables, we found that the climatic variable that best fit the quadratic model for *D*. *frontalis* was Vapor Pressure Deficit (VPD, Table 3, Fig 3). Larger values of VPD were associated with greater abundances of *D*. *frontalis* per trap per year, until declining somewhat at the highest VPD values (Fig 3). Since VPD increases nonlinearly with warmer temperatures, these data show how *D*. *frontalis* is thus highly temperature dependent.

**Table 3:** Mixed model analysis for total year abundance of *Dendroctonus frontalis* per trap. Akaike Information Criterion (AIC, Akaike, 1973), estimated parameters, contribution to total variance (of random terms) and significance for the selected mixed model are indicated.

Parameter or source of variation	Estimate	%^¶^	*P*
Fixed effects			
AIC	2015.7		
Intercept	-85746		0.0140
Vapor Pressure Deficit annual mean	209672		0.0001
(Vapor Pressure Deficit annual mean)^2^	-118044		< .0001
*Random effects*			
*State*	690427	1.9	0.4650
*Transect(State)*	18705784	52.3	0.0538
*Residual*	16356227	45.7	

^¶^ Percent contribution to total variance (where 100% is the sum of variances of all random terms).

**Fig 4:**
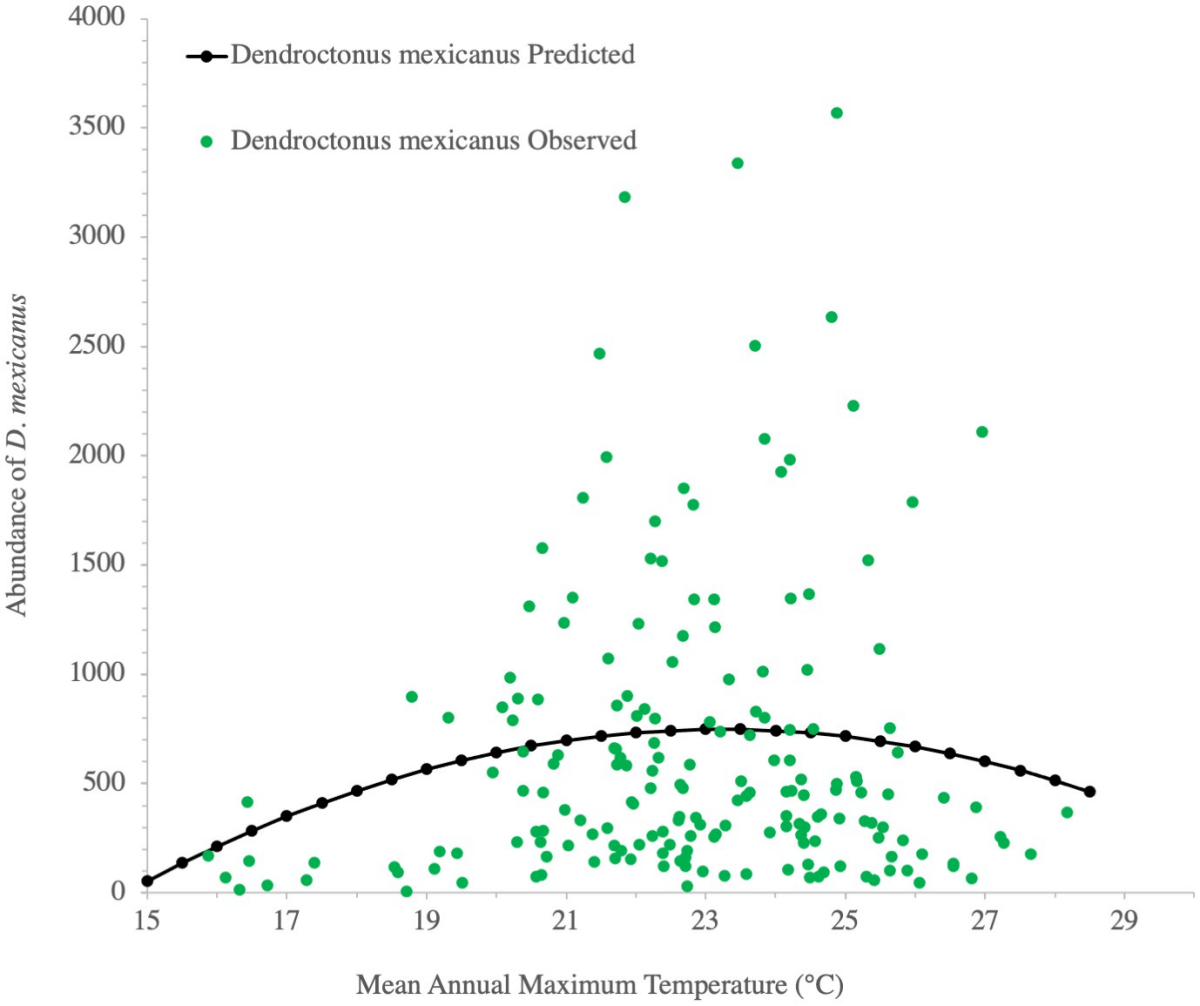
Abundance (total per year per trap) of *Dendroctonus mexicanus* against Mean Annual Maximum Temperature (MMAX, °C). Predicted data are estimated solely from the fixed terms of a quadratic response function in a mixed model, as in Table 3.

In contrast, for *D*. *mexicanus*, VPD was not the most important climatic variable explaining its abundance. The best mixed model considered Mean Annual Maximum Temperature and the highest peak of *D*. *mexicanus* abundance occurred between 20°C and 26°C (Fig 4, Table 4). Maximum temperatures either below 20°C or above 26°C had lower abundance of *D*. *mexicanus*.

**Table 4:** Mixed model analysis for total year abundance of *Dendroctonus mexicanus* per trap. Akaike Information Criterion (AIC, Akaike, 1973), estimated parameters, contribution to total variance (of random terms) and significance for the selected mixed model are indicated.

Parameter or source of variation	Estimate	%^¶^	*P*
Fixed effects			
AIC	3261.5		
Intercept	-5177.4		0.0145
Mean Maximum Annual Temperature	504.9		0.0003
(Mean Maximum Annual Temperature)^2^	-10.9		0.0004
*Random effects*			
*State*	64598	15.7	0.1627
*Transect(State)*	81101	19.7	0.0584
*Residual*	266037	64.6	

^¶^ Percent contribution to total variance (where 100% is the sum of variances of all random terms).

State and Transect nested in State as sources of variation and as random effects were not significant (P > 0.05) for both *Dendroctonus* species (Tables 3 and 4).

We also explored grouping States as Regions (as random effect), where (same grouping as in Table 1): Northern Region = Chihuahua, Durango, Coahuila and Nuevo León; Central Region = Jalisco, Michoacán, State of México, Hidalgo and Querétaro; Southern Region = Oaxaca and Chiapas. Transects were also nested in Regions. For both cases, the random effects of Regions and Transects were not significant (P > 0.05) for the abundance of *D*. *frontalis* as response of Vapor Pressure Deficit, and for *D*. *mexicanus* as response of Mean Annual Maximum Temperature.

The plotting of Mean Annual Temperatures from the center of each transect for the period 1958–2021, reveals that the studied years 2015–2016 were not atypical: they were within the longer-term trend of a steady increase of temperatures, evident after 1980 (S2 Fig).

## 4. Discussion

### 4.1 Relationship between climate variables and bark beetle abundance

The abundance of bark beetle flying populations was related to climate variables: Vapor Pressure Deficit and Mean Annual Temperature for *D*. *frontalis*, and Mean Maximum Annual Temperature and Mean Annual Temperature for *D*. *mexicanus*. The *D*. *frontalis* abundance pattern clearly shows that abundance of this beetle is highest at sites with a Mean Annual Temperature (MAT) of 18°C (Fig 2) and Vapor Pressure Deficit ≥ 1 (Fig 3). Vapor Pressure Deficit can be seen as a proxy indicator of atmospheric drought [72]. Rising VPD has been linked to declining (more negative) water potential inside trees [80], mechanistically linking increasing VPD with physiological drought stress in plants [81]. Those two variables (MAT and VPD) that promote drought are also known to be important for bark beetle outbreaks in xeric habitats [82,83], although such association with specifically VPD have not been proven previously in Mexico. During the warm dry season (March-May) lower altitudes experience higher temperatures in the center of the Transverse Volcanic Belt in Mexico—the same region and elevation (between 600 and 1500 m a.s.l.) where the main distribution of *D*. *frontalis* occurs in the country [8]. Our results are similar to findings in the Sierra Gorda mountains of Querétaro state by López-Gómez *et al*. [84], which found that *D*. *frontalis* abundance was positively correlated with temperatures and temperature/precipitation ratio (a climate variable that, like VPD, expresses the balance between temperature and precipitation).

For *D*. *mexicanus*, the highest relative abundances occur at a lower Mean Annual Temperature than for *D*. *frontalis* (Fig 2). This is consistent with the fact that *D*. *mexicanus* occurs at higher altitudes, with its main distribution range along the Transverse Volcanic Belt and between 2000 and 2500 m a.s.l. [8]. Our data indicate that a Mean Maximum Annual Temperature between 20 and 26°C correlates with higher abundances of *D*. *mexicanus*, as shown in Fig 4 (notice that *D*. *mexicanus* occupy a climatic space colder than *D*. *frontalis*, because Mean Maximum Annual Temperatures are not fully comparable with the Mean Annual Temperatures, since the former have overall by far higher values than the later). Our results are also similar to findings by López-Gómez *et al*., [84], where higher temperatures were associated with a higher abundance of flying *D*. *mexicanus* in the mountains of Querétaro. VPD was not the most important climatic variable for *D*. *mexicanus* abundance, perhaps because this species occurs at higher elevations than *D*. *frontalis*, where there is a narrower range of VPD values. Note that another study with a bark beetle species occurring in cool boreal forests found that they are not responsive to climatic moisture deficit [85]; this hypothesis warrants further investigation. Also, it is worth considering that the semiochemicals used in this study were designed and tested for monitoring *D*. *frontalis* [86]. We included *D*. *mexicanus* in our analyses because it consistently appeared in our traps, but we cannot ensure that the semiochemical combination and concentrations were optimal for *D*. *mexicanus*. Our conclusions in this species should therefore be considered preliminary, and further detailed investigation is needed on the level of attraction produced in this species by the semiochemicals used.

Our findings of a strong relationship between weather and *Dendroctonus* species distribution are consistent with the results of Moser *et al*., (2005) from pheromone-baited traps for *Dendroctonus* species in Arizona (the northern distribution limit for *D*. *mexicanus*). They reported that maximum temperatures during bark beetle flight fluctuate from 19°C in winter to approximately 38°C in summer. In our study, captures of *D*. *mexicanus* individuals dropped at temperatures below 16°C, and even *D*. *frontalis* individuals were less active at cold temperatures [87].

The large dispersion of data (of number of individuals trapped per site and year) for the same intervals of VPD and Annual Mean Maximum temperatures, noticeable of Figs 3 and 4, appears to be a feature common on this type of sampling, where for the same climatic interval where average insect abundance is higher, it is possible to have either very low or very high number of individuals trapped at individual traps. See for example the similarity of our trends in Figs 3 and 4 with the dispersion data from Moser et al., [85] on their Fig 4 (*Dendroctonus frontalis*) and Fig 3 (*D*. *mexicanus*). That data dispersion indicates that there are other local factors, additional than climate, that might be triggering the increase or decrease of *Dendroctonus* flying populations.

It is important to stress that our study provide evidence of the climatic intervals on which flying populations of *Dendroctonus frontalis* and *D*. *mexicanus* occur at locations southern that the ones reported for most of the comparable available literature for the genus *Dendroctonus*, generated mostly at USA and Canada.

The fact that both sources of variation, State or Transect nested in Sate, were not significant, indicates that most of the climatic effects expressed at State and Transect were likely absorbed first by the climatic fixed terms of the model. In other words, State and Transects nested in States should be viewed as sources of variations that contain factors of variation other than climatic effects. The large significance of the climatic fixed terms and the lack of significance of States and Transects as random effects, indicate that the abundance of the two species of *Dendroctonus* is influenced by some specific intervals of climatic values, independently if those climates occurring at the Northern Mexican states (as Chihuahua, Durango, Coahuila or Nuevo León), or at the Southern states (as Oaxaca or Chiapas).

### 4.2 Possible consequences under future climatic change scenarios

Our results indicate that if temperature continues to increase due to climate change, abundances of *D*. *frontalis* will likely continue to increase in numbers at lower elevations (at least until temperatures surpass their optimum) and/or move to higher elevations as their optimal conditions are reached at higher altitudes that were previously too cold for them, as reported for other species under climate change conditions [32,88]. Consequently, these species may pose an increasingly serious threat to the health of Mexican pine forests, especially at their low-elevation xeric limits (generally the extreme lower limit of the natural distribution of each tree species, *sensu* Mátyás [89]). From our data, we infer that since *D*. *mexicanus* has a large area of sympatry with *D*. *frontalis*, it would likely also move to higher elevations. Our data do not rule out the possibility that warming temperatures might exceed the optimum values for these insects, and thus their populations might decline beyond that warmer temperature threshold; however, those higher temperatures could also be deleterious to their host tree populations. For example, other studies of insect-plant interactions have documented that temperatures above herbivores’ thermal limits can decrease their populations directly, at the same time as extreme temperatures may decrease plant quality for herbivores and therefore decrease herbivore performance indirectly as well [90].

The amount of moisture available for the trees also seems to play a role in the abundance of bark beetles in general. As atmospheric drought stress intensifies with warming temperatures and rising VPD, tree water potentials decline, weakening the trees and inhibiting their defense responses [15,25,81]. This may be linked to an increase in emission of volatile organic compounds, which are known to play important roles in signaling and aggregation of insects [91,92]. Likely the trees are weakened and thus easier attacked by the bark beetles [81].

For both bark beetle species, it is likely that as temperature increases and precipitation decreases due to climatic change [93], they will follow their optimal temperature ranges, shifting their most serious outbreaks to higher-elevation forests than presently observed. Several studies have also found that Scolytinae abundance is related to higher ambient temperatures, which coincides with the lower altitude interval of its distribution [39,94,95].

As stated by Biederman *et al*. [28], the drivers of bark beetle population irruptions and crashes are still not fully understood. These beetle population dynamics are apparently driven by complex interactions among tree host abundance and susceptibility, beetle density, weather, symbiotic associations, and natural enemies, with all these interactions affecting the potential of bark beetles to function as major agents of widespread forest disturbance [11]. Thus, further research is needed on these biotic interactions with bark beetle populations, in addition to associations with climatic drivers.

To extrapolate these correlation patterns between bark beetle abundance and temperature, we should also consider how pine hosts may or may not shift their current distributions in future climates [24]. One scenario is that these pine populations stay where they currently are distributed, and therefore host pines would be more attacked in sites where temperature is optimal for bark beetles. If these attacks are sufficiently severe and recurrent, forest populations may tend towards local extinction at those sites. Also, it has been reported that naïve pine populations (not exposed to bark beetles previously) are less able to defend themselves, and therefore may be more susceptible to *Dendroctonus* attack (reviewed by Raffa *et al*. [96]). A hypothetical alternative situation is that host pine species start to shift their distributions in response to climate change and bark beetles follow them without causing major distress. However, this seems unlikely because the natural migration speed of forest tree species is much slower than the migration rate projected to be necessary to keep these tree species coupled to the climate conditions to which they are adapted [97–99]. A third scenario is that once preferred pines disappear from a particular site, the resident bark beetles will colonize new pine species. This is more complicated and uncertain, since the ability to colonize a specific pine host has been linked with bark beetle fungal symbionts [100,101]. Of course, these hypothetical scenarios need to be taken with caution, since more sophisticated, predictive, site-specific models considering age, species composition and structure of the stands, are needed for a more robust prediction.

### 4.3 Limitations of this research

There is a large body of biological and ecological information on *D*. *frontalis* (Coulson and Klepzig [102] and references therein). This includes a particularly wide breadth of knowledge on its pheromonal communication system. For *D*. *mexicanus*, on the other hand, information is extremely limited [44]. The pheromone blend used in our work was designed for *D*. *frontali* [44,86]. However, it is also reported to attract individuals of *D*. *mexicanus* in Mexico and the United States [46–50,52,87,103]. Therefore, we are confident that the conclusions we have reached about the relative abundance of the trapped *D*. *frontalis* numbers in this study are solid, but we are somewhat more cautious in our interpretations concerning *D*. *mexicanus*. Although more confident interpretations for trap data on *D*. *mexicanus* relative abundance will be obtained someday when an appropriate species-specific pheromone blend becomes available and when the basic ecology of this species is better described, the nationwide study reported here certainly advances our knowledge of *D*. *mexicanus* distribution and abundance.

This determination of how climate contributes to the dynamics of these two important *Dendroctonus* species is a useful advance but represents only an initial understanding of what are likely very complex drivers of both the distribution and abundance of *Dendroctonus* populations as climate change unfolds. Further developments will require more sophisticated models that consider specific characteristics of current forest stands (e.g., pine species present, basal area, and recent history of growth trends and beetle outbreaks) and account for potential shifts in host tree distributions [104–107]. However, such finely parameterized models may only prove effective at local or regional scales, which highlights the importance of less complex, broad-scale empirical models like the one used in this study.

Monitoring bark beetle populations using pheromone-baited traps (as per Hayes *et al*. [108]) proved to be an effective approach to collect beetle population data for this study, as this approach provided reliable information on variation in species presence and abundance across elevational gradients using trap transects that sampled flying beetles. However, Hayes *et al*. [108] also indicate an inconsistent relationship between flying beetle population data and concurrent host tree mortality. In other words, sampling flying beetles at the same time as tree mortality may underestimate the impacts of beetles on trees because processes like tree mortality often lag significantly behind beetle flights and infestations. Thus, long-term monitoring like the successful *D*. *frontalis* monitoring system in the Southern United States may be required [43]. In our sites, the correlation between bark beetle abundance and tree infestation was not recorded; these relationships need to be addressed in future studies.

### 4.4 Future research needs

From a different perspective, bark beetles can be considered an ecosystem component that promotes the renewal of aging stands, but this role needs to be better understood in the context of climatic change. The number and intensity of outbreaks are undoubtedly increasing due to debilitated trees because hotter droughts are occurring due to climate change [21,22]. Unfortunately, at the same time, in the case of the main mountain systems in México, recruitment via natural regeneration has been more difficult because the March-May warm dry season is becoming ever warmer and drier due to climate change [109]. Such a combination of processes poses an important challenge for both commercial forest management and for the conservation of Natural Protected Areas to achieve stand renewal after bark beetle outbreaks.

There is an important body of data accumulating in recent years, that indicates an association of increasing annual temperatures and hotter drought periods with a larger number of bark beetles outbreaks that need a sanitary intervention by logging. In our view, this is clear evidence of the association between the ongoing climatic change and the increase of bark beetle populations [21]. However, a nation-wide monitoring effort is needed to trap bark beetle on sites with disparate climatic characteristics, as we have presented here, but for longer periods of time. That in order to have a stronger support of the association between increasing temperatures and drought periods with the occurrence of more abundant bark beetle flying populations.

## 5. Conclusions

1. Bark beetles appear to have optimal temperature conditions: between 17°C and 20°C of Mean Annual Temperature for *Dendroctonus frontalis* and two optimal intervals for *D*. *mexicanus*: between 11°C and 13°C and between 15°C and 18°C.
2. Drought conditions seem to favor increases in bark beetle populations, as suggested by the association between higher *D*. *frontalis* abundance and higher Vapor Pressure Deficit values.
3. Because environmental conditions are shifting due to human-caused climate change, and historical climate for a given site is progressively occurring at higher altitudes in mountain regions, it might be possible that bark beetles will likely follow their optimal climate conditions by shifting to higher altitudes.
4. Given the projected increase of temperatures and decrease of precipitation for Mexican continental regions due to climatic change, and considering the association between high values of vapor pressure deficit and values of mean maximum annual temperatures with the abundance of *D*. *frontalis* and *D*. *mexicanus*, respectively, it seems likely that bark beetle populations will increase along with the increase of temperatures and decrease of precipitation, which may lead to worsening infestations and affect pine fitness.

## Supporting information

S1 Fig Total abundance of *Dendroctonus* captured by trap and per year and summed by interval of 1°C of Mean Annual Temperature for (a) *Dendroctonus frontalis*, and (b) *D*. *mexicanus*. Notice the *X* axis of temperature is the same for the two species, to facilitate comparison of the climatic space, but the *Y*-axis has a very different scale, depending on the abundance of each species.
(TIFF)

S2 Fig Trend of the Mean Annual Temperatures (MAT) from the center of each transect for the period 1958–2021. Average (across the center of all altitudinal transects) of MAT (light blue dots) is connected by a line to show annual variability (light blue line) and by a 10-year trendline (loess, span = 10, solid black line) to show the overall trend. Average across transects of the studied 2015–2016 years are highlighted as large red dots. Background gray dots are the MAT for the center of each transect (the coldest and warmest transects are omitted here due to Y-axis scale reasons, although their values were included to obtain the overall yearly averages). Vertical bars are 95% confidence interval in the all-transect annual MAT values. Data from TerraClimate website.
(TIF)

S1 File S1 to S11 Tables (one Table per state), with details of transects (coordinates and altitude), sorted by state, from North to South and West to East. (DOCX)

## References

[1] Farjon A., Styles B., 1997. Flora Neotropica. Monograph 75. Pinus (Pinaceae). Organization for Flora Neotropica. New York Botanical Garden, New York. doi: 10.1007/bf02860793

[2] Zúñiga G., Cisneros R., Hayes J. L., Macías Sámano J., 2002b. Karyology, geographic distribution and origin of the genus Dendroctonus Erichson (Coleoptera: Scolytidae). Ann. Entomol. Soc. Am. 95: 267–275.

[3] Salinas-Moreno Y., Mendoza M. G., Barrios M. A., Cisneros R., Macías-Sámano J., Zúñiga G., 2004. Areography of the genus Dendroctonus (Coleoptera: Curculionidae: Scolytinae) in Mexico. J. Biogeo. 31, 1163–1177. doi: 10.1111/j.1365-2699.2004.01110.x

[4] Wood S. L., 1982. The Bark Ambrosia Beetles of North and Central America (Coleoptera: Scolytidae): a taxonomic monograph. Great Basin Nat. 6: 1–1359.

[5] Salinas-Moreno Y., Ager A., Vargas C.F., Hayes J.L., Zúñiga G. 2010b. Determining the vulnerability of Mexican pine forests to bark beetles of the genus Dendroctonus Erichson (Coleoptera: Curculionidae: Scolytinae). Forest Ecol. Manage. 260,52–61. doi: 10.1016/j.foreco.2010.03.029

[6] Six D. L., Bracewel R., 2015. Dendroctonus, in Vega F.E., Hofstetter R.W. (Eds.), Bark Beetles, Biology and Ecology of Native and Invasive Species. Elsevier, CA, USA. pp. 305–350.

[7] Armendáriz-Toledano F., Zúñiga G., 2017. Illustrated key to species of genus Dendroctonus (Coleoptera: Curculionidae) occurring in Mexico and Central America. J. Insect. Sci. 17, 1–15. doi: 10.1093/jisesa/iex009 28355476 PMC5416895

[8] Salinas-Moreno Y., Vargas-Mendoza C.F., Zúñiga G., Víctor J., Ager A., Hayes J.L., 2010a. Atlas de distribución geográfica de los descortezadores del género Dendroctonus (Curculionidae: Scolytinae) en México. Instituto Politécnico Nacional IPN, Comisión Nacional Forestal CONAFOR, Cd. de México, Mexico. 90 p.

[9] Safranyik, L., Wilson L. 2006. The mountain pine beetle: a synthesis of biology, management and impacts on lodgepole pine. Canadian Forest Service, Pacific Forestry Centre, Natural Resources Canada. Victoria, Canadá. 10.1016/0378-1127(80)90040-7.

[10] Guldin J.M. 2011. Silvicultural Considerations in Managing Southern Pine Stands in the Context of Southern Pine Beetle. In Southern Pine Beetle II; Gen. Tech. Rep. SRS-140; Coulson R.N., Klepzig K.D., Eds.; U.S. Department of Agriculture Forest Service, Southern Research Station: Asheville, NC, USA, pp. 317–352. doi: 10.2737/srs-gtr-140

[11] Raffa K.F.B., Aukema B.H., Bentz B.J., Carroll A.L., Hicke J.A., Turner M.G., et al, 2008. Cross-scale drivers of natural disturbances prone to anthropogenic amplification: The dynamics of bark beetle eruptions. BioScience 58(6):501–517. doi: 10.1641/b580607

[12] Trumbore S., Brando P. and Hartman H. 2015. Forest health and global change. Science 349: 819–822. 26293952 10.1126/science.aac6759

[13] Ryan M.G., Sapes G., Sala A., Hood S.M., 2015. Tree physiology and bark beetles. New Phytol. 205, 955–957. doi: 10.1111/nph.13256 25580650

[14] Byers J. A., 1995. Host-tree chemistry affecting colonization in bark beetles, in Cardé R. T., Bell W. J. (Eds.), Chemical ecology of insects 2, Chapman and Hall, New York. pp. 154–213. doi: 10.1007/978-1-4615-1765-8_5

[15] Borden J, H., 1982. Aggregation pheromones, in Mitton J. B., Sturgeon K. B. (Eds.), Bark beetles in North American conifers, a system for the study of evolutionary biology, University of Texas Press. Austin, Texas, USA. pp. 75–139.

[16] Evenden M.L., Whitehouse C.M., Sykes J., 2014. Factors influencing flight capacity of the mountain pine beetle (Coleoptera: Curculionidae: Scolytinae). Environ. Entomol. 43, 187–196. doi: 10.1603/EN13244 24367930

[17] Bentz B., Régnière J., Fettig C.J., Hansen E.M., Hayes J.L., Hicke J.A., et al, 2010. Climate change and bark beetles of the western United States and Canada: direct and indirect effects. Bio Sci. 60, 602–613. doi: 10.1525/bio.2010.60.8.6

[18] Allen C.D., Macalady A.K., Chenchouni H., Bachelet D., McDowell N., Vennetier M., et al, 2010. A global overview of drought and heat-induced tree mortality reveals emerging climate change risks for forests. Forest Ecol. Manage. 259, 660–684. doi: 10.1016/j.foreco.2009.09.001

[19] Allen C. D., Breshears D. D., McDowell N. G., 2015. On underestimation of global vulnerability to tree mortality and forest die-off from hotter drought in the Anthropocene. Ecosphere 6, 129. doi: 10.1890/es15-00203.1

[20] Breshears D.D., Fontaine J.B., Ruthrof K.X., Field J.P., Feng X., Burger J.R., et al. (2021), Underappreciated plant vulnerabilities to heat waves. New Phytol, 231: 32–39. doi: 10.1111/nph.17348 33728638

[21] Gómez-Pineda E., Hammond W. M., Trejo-Ramirez O., Gil-Fernández M., Allen C. D., Blanco-García A., et al. 2022. Drought years promote bark beetle outbreaks in Mexican forests of Abies religiosa and Pinus pseudostrobus. Forest Ecology and Management 505: article 119944:1–11. doi: 10.1016/j.foreco.2021.119944

[22] Hammond W. M., Williams A. P., Abatzoglou J. T., Adams H. D., Klein T., López Rodríguez R., et al. 2022. Global field observations of tree die-off reveal hotter-drought fingerprint for Earth’s forests. Nature Communications 13, 176. doi: 10.1038/s41467-022-29289-2 35383157 PMC8983702

[23] Weed A.S., Ayres M.P., Hicke J.A., 2013. Consequences of climate change for biotic disturbances in North American forests. Ecol. Monogr. 83, 441–470. doi: 10.1890/13-0160.1

[24] Alfaro R.I., Fady B., Vendramin G.G., Dawson I.K., Fleming R.A., Sáenz-Romero C., et al, 2014. The role of forest genetic resources in responding to biotic and abiotic factors in the context of anthropogenic climate change. Forest Ecol. Manage. 333, 76–87. doi: 10.1016/j.foreco.2014.04.006

[25] Kolb T.E., Fettig C.J., Ayres M.P., Benz B.J., Hicke J.A., Mathiasen R., et al, 2016. Observed and anticipated impacts of drought on forest insects and diseases in the United States. Forest Ecol. Manage. 380, 321–334. doi: 10.1016/j.foreco.2016.04.051

[26] Sáenz-Romero C., Mendoza-Maya E., Gómez-Pineda E., Blanco-García A., Endara-Agramont A.R., Lindig-Cisneros R., et al, 2020. Recent evidence of Mexican temperate forest decline, need for ex situ conservation, assisted migration and translocation of species ensembles as an adaptive management to face projected climatic change impacts in to megabiodiverse country. Canadian J Forest Res. 50, 843–854. doi: 10.1139/cjfr-2019-0329

[27] Hart S.J., Veble T.T., Eisenhart K.S., Jarvis D., Kulakowski D., 2014. Drought induces spruce beetle (Dendroctonus rufipennis) outbreaks across northwestern Colorado. Ecology 95, 930–939. doi: 10.1890/13-0230.1 24933812

[28] Biederman P.W., Müller J., Grégoire J.C., Gruppe A., Hagge J., Hammerbacher A., et al, 2019. Bark Beetle Population Dynamics in the Anthropocene: Challenges and Solutions. Trends Ecol. Evol. 34, 914–924. doi: 10.1016/j.tree.2019.06.002 31262532

[29] Ning H., Tang M., Chen H., 2021. Impact of climate change on potential distribution of chinese white pine beetle Dendroctonus armandi in China. Forests 12, 544. doi: 10.3390/f12050544

[30] Bentz B.J., Vandygriff J.C., Jensen C., Coleman T., Maloney P., Smith S., et al, 2014. Mountain pine beetle voltinism and life history characteristics across latitudinal and elevational gradients in the western United States. Forest. Sci. 60, 434–449. doi: 10.5849/forsci.13-056

[31] Dhar A., Parrott L., Hawkins C.D.B., 2016. Aftermath of Mountain Pine Beetle outbreak in British Columbia: stand dynamics, management response and ecosystem resilience. Forests 7, 171. doi: 10.3390/f7080171

[32] Tran J.K., Ylioja T., Billings R.F., Regniere J., Ayres M.P., 2007. Impact of minimum winter temperatures on the population dynamics of Dendroctonus frontalis. Ecol. Appli. 17, 882–899. doi: 10.1890/06-0512 17494404

[33] Friedenberg N. A., Powell J. A., Ayres M. P., 2007. Synchrony’s double edge: Transient dynamics and the Allee effect in stage structured populations. Ecol. Lett., 10: 564–573. doi: 10.1111/j.1461-0248.2007.01048.x 17542935

[34] Jönsson A.M., Appelberg G., Harding S., Bärring L. 2009. Spatiotemporal impact of climate change on the activity and voltinism of the spruce bark beetle, Ips typographus. Global Change Biology 15, 486–499. doi: 10.1111/j.1365-2486.2008.01742.x

[35] Hansen E.M.; Bentz B.J. 2003. Comparison of reproductive capacity among univoltine, semivoltine, and re-emerged parent spruce beetles (Coleoptera: Scolytidae). Canadian Entomology 135, 697–712. doi: 10.4039/n02-109

[36] Cervantes-Martínez R., Cerano-Paredes J., Sánchez-Martínez G., Villanueva-Díaz J., Esquivel-Arriaga G., Cambrón-Sandoval V. H., et al, 2019. Historical bark beetle outbreaks in Mexico, Guatemala and Honduras (1895–2015) and their relationship with droughts. Revista Chapingo Serie Ciencias Forestales y del Ambiente, 25(2), 269–290. doi: 10.5154/r.rchscfa.2019.01.006

[37] SINIARN., 2020. Sistema Nacional de Información Ambiental y de Recursos Naturales (https://apps1.semarnat.gob.mx:8443/dgeia/compendio_2020/dgeiawf.semarnat.gob.mx_8080/ibi_apps/WFServlete3bc.html). Accessed April 20th 2021.

[38] Rivera-Rojas M., Lucatelli B., Billings R. 2010. Climate change and outbreaks of Southern Pine Beetle Dendroctonus frontalis in Honduras. Forest Systems 19(1):70–76. doi: 10.5424/fs/2010191-01168

[39] Rubín-Aguirre A., Saenz-Romero C., Lindig-Cisneros R., del-Rio-Mora A. A., Tena-Morelos C. A., Campos-Bolaños R., et al, 2015. Bark beetle pests in an altitudinal gradient of to Mexican managed forest. Forest Ecol. Manage. 343:73–79. doi: 10.1016/j.foreco.2015.01.028

[40] Jactel H., Koricheva J., & Castagneyrol B. 2019. Responses of forest insect pests to climate change: not so simple. Current opinion in insect science, 35, 103–108. doi: 10.1016/j.cois.2019.07.010 31454625

[41] Turchin P., Lorio P.L. Jr., Taylor A.D., Billings R.F., 1991. Why do populations of southern pine beetles (Coleoptera: Scolytidae) fluctuate? Environ. Entomol. 20, 401–409. doi: 10.1093/ee/20.2.401

[42] Pureswaran D. S., Roques A., & Battisti A. 2018. Forest insects and climate change. Current Forestry Reports, 4(2), 35–50. doi: 10.1007/s40725-018-0075-6

[43] Billings R.F., Upton W.W. 2010. A methodology for assessing annual risk of southern pine beetle outbreaks across the southern region using pheromone traps, in, Pye J. M., Rauscher H., M., Sands Y., Lee D. C., Beatty J. S. (Eds.), Advances in threat assessment and their application to forest and rangeland management, General Technical Report PNW-GTR-802. Portland, OR: U.S. Department of Agriculture, Forest Service, Pacific Northwest and Southern Research Stations. Vol 1. pp. 73–85. doi: 10.2737/pnw-gtr-802

[44] Macías-Sámano, J.E., Niño, A., 2016. Protocolo para monitoreo de descortezadores de coníferas mediante el uso de atrayentes y semioquímicos, para México y Centroamérica. El Colegio de la Frontera Sur, ECOSUR y el Programa Internacional del Servicio Forestal de los Estados Unidos, USFS-IP. 48 p. 10.29104/phi-aqualac/2017-v9-2-04.

[45] Cibrián-Tovar, D., Méndez, J. T., Campos, R., Yates III, O., Flores, J. 1995. Insectos Forestales de México/Forest Insects of México. Universidad Autónoma Chapingo, SARH, USDA, Natural Resources Canada, Comisión Forestal de América del Norte. FAO. 10.5154/r.rchscfa.2015.09.043.

[46] Sánchez-Martínez G., Reséndiz-Martínez J.F., 2020. Respuesta de Dendroctonus frontalis Zimmerman y Dendroctonus mexicanus Hopkins a dos atrayentes semioquimicos en la Sierra Gorda de Queretaro, Mexico. Southwestern Entomol. 45, 511–520 doi: 10.3958/059.045.0219

[47] Avilés-Carrillo I., Vergara-Pineda S., Cambrón-Sandoval V.H., Obregón-Zúñiga A., 2016. Fluctuación poblacional de Dendroctonus frontalis Zimmermann, 1868 y Dendroctonus mexicanus Hopkins, 1909 (Curculionidae: Scolytinae) en relación a la variación en la altitud y factores climáticos en un bosque de pino en Zimapán, Hidalgo. Entomología Mexicana 3, 649–655. doi: 10.21829/azm.2018.3412141

[48] Hernández-Muñoz G., Soto-Correa J.C., Cambrón-Sandoval V.H., Avilés-Carrillo I., 2017. Explosión de la abundancia de descortezadores, un acontecimiento adelantado a la primavera en el bosque de pino en Hidalgo. Entomol. Forest. Mex. 4, 525–530.

[49] Soto-Correa J.C., Avilés-Carrillo I., Girón-Gutiérrez D., Cambrón-Sandoval V.H., 2019. Abundancia altitudinal de Dendroctonus frontalis (Coleóptera: Curculionidae) en relación a variables climáticas en Hidalgo, México. Rev. Biol. Trop.l 67, 370–379.

[50] Soto-Correa J. C., Girón-Gutiérrez D., Cambrón-Sandoval V. H., 2020. Coloración y abundancia de Dendroctonus mexicanus Hopkins, 1905 en cuatro regiones de México. Rev. Mex. Ciencias Forest.11,163–184. doi: 10.29298/rmcf.v11i59.668

[51] Armendáriz-Toledano F., Zúñiga G., García-Román L. J., Valerio-Mendoza O., García-Navarrete P. G., 2018. Guía ilustrada para identificar a las especies del género Dendroctonus presentes en México y Centroamérica. Instituto Politécnico Nacional. CDMX, México. 119 p.

[52] Morales-Rangel A., Cambrón-Sandoval V.H., Jones W.R., Obregón-Zúñiga. 2018. Efecto de la temperatura en poblaciones de Dendroctonus frontalis Zimmerman y Dendroctonus mexicanus Hopkins (Coleóptera: Curculionidae: Scolytinae) bajo un escenario de cambio climático en la Sierra Gorda queretana. Acta Zoológica Mexicana 34, e3412141. doi: 10.21829/azm.2018.3412141

[53] Sáenz-Romero C., Rehfeldt G. E., Crookston N. L., Pierre D., St-Amant R., Bealieau J., et al. 2010. Contemporary and projected spline climate surfaces for Mexico and their use in understanding climate-plant relationships. Climatic Change, 102, 595–623.

[54] CCAD (Comisión Centroamericana del Ambiente y Desarrollo). 2017. Estrategia Regional de Salud y Sanidad Forestal para Centroamérica y República Dominicana 2016–2026. CCAD, FAO, Panamá. 91 p.

[55] Macías-Sámano J.E., Ortiz P., García Ochaeta J.F y Masaya L. 2021. Los insectos descortezadores de los pinos de Guatemala. Biología, ecología y manejo en la salud y la sanidad forestal. USFS-IP y INAB, Guatemala. 88 p.

[56] Pérez Miranda R., González Hernández A., Velasco Bautista E., Romero Sánchez M. E., Arriola Padilla V. J., Acosta Mireles M., et al. (2021). Análisis temporal de la distribución de Dendroctonus mexicanus Hopkins (1905) en México (2009–2018). Revista Mexicana de Ciencias Forestales, 12(67), 27–55. doi: 10.29298/rmcf.v12i67.1079

[57] Lauer W. 1978. Timberline studies in central Mexico. Artic Alpine Res. 10: 383–396. doi: 10.2307/1550769

[58] Fabián-Plesníková I., Sáenz-Romero C., Terrazas T., Reyes-Ramos A., Martínez-Trujillo M., Cruz-De-León J., et al. 2022. Traumatic ducts size varies genetically and is positively associated to resin yield of Pinus oocarpa open-pollinated progenies. Silvae Genetica 71(1):10–19. doi: 10.2478/sg-2022-0002

[59] Sáenz-Romero C, Rehfeldt GE, Ortega-Rodríguez JM, Marín-Togo MC, Madrigal-Sánchez M. 2015. Pinus leiophylla suitable habitat for 1961–1990 and future climate. Botanical Sciences 93 (4): 709–718. doi: 10.17129/botsci.86

[60] López-Upton J. 2002. Pinus pseudostrobus Lindl. In: Tropical Tree Seed Manual. Vozzo J A (ed). USDA Forest Service. Pp: 636–638.

[61] Donahue J. K., & López-Upton J. 1996. Geographic variation in leaf, cone and seed morphology of Pinus greggii in native forests. Forest Ecology and Management, 82(1–3), 145–157. doi: 10.1016/0378-1127(95)03677-6

[62] Perry J. P. Jr 1991. The pines of Mexico and central America. Timber Press, Inc.

[63] Sánchez-González A. 2008. Diversity and distribution of Mexican pines, an overview. Madera y Bosque. 14(1): 107–120.

[64] Sáenz-Ceja J E, Arenas-Navarro M, Torres-Miranda A. 2022. Prioritizing conservation areas and vulnerability analyses of the genus Pinus L. (Pinaceae) in Mexico. Journal for Nature Conservation 67(126171):1–15. doi: 10.1016/j.jnc.2022.126171

[65] Dvorak W. S.; Hodge G. R.; Kietzka J. E.; Malan F.; Osorio L. F.; Stanger T. K. 2000. Pinus patula. In: Conservation and testing of tropical and subtropical forest tree species by the CAMCORE Cooperative 2000 pp.148–173. Raleigh, NC, USA.

[66] Macías-Sámano J. E., Zúñiga G., 2016. Estado actual del conocimiento en Mexico sobre el uso de semioquímicos que median las interacciones entre insectos descortezadores y las coníferas. In: Anaya-Lang A.L., Espinosa F.J., Macías F., Reigosa M. (Eds.), Ecología Química y Alelopatía: avances y aplicaciones, UNAM, México.

[67] Vidal-Zepeda R. 2005. Las regiones climáticas de México. Universidad Autónoma de México/Instituto de Geografía, México city, México. 212 p.

[68] SAS Institute Inc., 2004. SAS/STAT Computer Software. Release 9.1. 3rd Edition. SAS Institute Inc, Cary, North Carolina, USA.

[69] Gely C., Laurance S.G.W., Stork N.F., 2020. How do herbivorous insects respond to drought stress in trees? Biol. Rev. 95: 434–448. doi: 10.1111/brv.12571 31750622

[70] Huang J., Kautz M., Trowbridge A.M., Hammerbacher A., Raffa K.F., Adams H.D., et al, 2020. Tree defence and bark beetles in a drying world: carbon partitioning, functioning and modelling. New Phytol. 225, 26–36. doi: 10.1111/nph.16173 31494935

[71] Jaworski T., Hilszczański J., 2013. The effect of temperature and humidity changes on insect development their impact on forest ecosystems in the expected climate change. Forest Res. Papers 74, 345–355. doi: 10.2478/frp-2013-0033

[72] Abatzoglou J.T., Dobrowski S.Z., Parks S.A., Hegewisch K.C., 2018. TerraClimate, a high-resolution global dataset of monthly climate and climatic water balance from 1958–2015. Sci Data 5, 170191. doi: 10.1038/sdata.2017.191 29313841 PMC5759372

[73] Leites L.P., Rehfeldt G.E., Robinson A.P., Crookston N.L., Jaquish B., 2012. Possibilities and limitations of using historic provenance tests to infer forest species growth responses to climate change. Nat. Resour. Modeling 25, 409–433, doi: 10.1111/j.1939-7445.2012.00129.x

[74] Leites L.P., Robinson A.P., Rehfeldt G.E., Marshall J.D., Crookston N.L., 2012. Height‐growth response to climatic changes differs among populations of Douglas‐fir: A novel analysis of historic data. Ecol. Appl. 22, 154–165, doi: 10.1890/11-0150.1 22471081

[75] Sáenz‐Romero C., Lamy J.B., Ducousso A., Musch B., Ehrenmann F., Delzon S., et al, 2017. Adaptive and plastic responses of Quercus petraea populations to climate across Europe. Glob. Chang. Biol. 23, 2831–2847. doi: 10.1111/gcb.13576 27885754 PMC5624497

[76] Sáenz-Romero C., Kremer A., Nagy L., Újvári-Jármay É., Ducousso A., Kóczán-Horváth A., et al, 2019. Common garden comparisons confirm inherited differences in sensitivity to climate change between forest tree species. PeerJ 7:e6213. doi: 10.7717/peerj.6213 30671299 PMC6338101

[77] Moore J.L., Remais J.V., 2014. Developmental models for estimating ecological responses to environmental variability: structural, parametric, and experimental issues. Acta Biotheorica 62, 69–90. doi: 10.1007/s10441-014-9209-9 24443079 PMC3973433

[78] Régnière J., Powell J., Bentz B., Nealis V., 2012. Effects of temperature on development, survival and reproduction of insects: Experimental design, data analysis and modeling. J. Insect Physiol. 58, 634–647. doi: 10.1016/j.jinsphys.2012.01.010 22310012

[79] Akaike H., 1973. Information theory and an extension of the maximum likelihood principle. In: Petrov B.N., & Csaki F. (Eds.). Proceedings of the 2nd International Symposium on Information Theory. Budapest, Hungary, Akademiai Kiado, pp. 267–281.

[80] López J., Way D. A., Sadok W., 2021. Systemic effects of rising atmospheric vapor pressure deficit on plant physiology and productivity. Global Change Biol. 27, 1704–1720. doi: 10.1111/gcb.15548 33683792 PMC8251766

[81] Grossiord C., Buckley T.N., Cernusak L.A., Novick K.A., Poulter B., Siegwolf R.T.W., et al, 2020. Plant responses to rising vapor pressure deficit. New Phytol. 226, 1550–1566. doi: 10.1111/nph.16485 32064613

[82] Gaylord M. L., Kolb T. E., Pockman W. T., Plaut J. A., Yepez E. A., Macalady A. K., … McDowell N. G., 2013. Drought predisposes piñon-juniper woodlands to insect attacks and mortality. New Phytol., 198, 567–578. doi: 10.1111/nph.12174 23421561

[83] Kolb T.E., Keefover-Ring K., Curr S.J., Hofstetter R., Gaylord M., Raffa K. F., 2019. Drought-mediated changes in tree physiological processes weaken tree defenses to bark beetle attack. J. Chem. Ecol. 45, 888–900. doi: 10.1007/s10886-019-01105-0 31493165

[84] López-Gómez V., Torres-Huerta B., Reséndiz-Martínez J. F., Sánchez-Martínez G.,& Gijón-Hernández A. R. (2017). Influence of climatic parameters on the population fluctuations of the complex Dendroctonus frontalis Zimmerman, 1868 and Dendroctonus mexicanus Hopkins, 1909. Rev. Mex. Cienc. Forestales 41.

[85] Pettit J. M., Voelker S. L., DeRose R. J. y Burton J. L., 2002. Spruce beetle outbreak was not driven by drought stress: Evidence from a tree-ring iso-demographic approach indicates temperatures were more important. Global Change Biology, 26(10): 5829–5843. doi: 10.1111/gcb.15274 32654317

[86] Moreno B., Macías J. Sullivan B., Clarke S. R., 2008. Field response of Dendroctonus frontalis (Coleoptera: Scolytinae) to synthetic semiochemicals in Chiapas, Mexico. J. Eco. Entomol 101, 1821–1825. doi: 10.1603/0022-0493-101.6.1821 19133462

[87] Moser J.C., Fitzgibbon B.A., Klepzig K.D., 2005. The Mexican pine beetle, Dendroctonus mexicanus: first record in the United States and co-ocurrence with the southern pine beetle Dendroctonus frontalis (Coleoptera: Scolytidae or Curculionidae: Scolytinae). Entomol. News 116, 235–243.

[88] Bentz B., Duncan J.P., Powell J.A., 2016. Elevational shifts in thermal suitability for mountain pine beetle population growth in a changing climate. Forestry 89, 271–283. doi: 10.1093/forestry/cpv054

[89] Mátyás C., 2010. Forecasts needed for retreating forests. Nature 469, 1271. doi: 10.1038/4641271a 20428144

[90] Stocks R., Verheyen J., Van Dievel M., Tüzün N. 2017. Daily temperature variation and extreme high temperatures drive performance and biotic interactions in a warming world Current Opinion in Insect Science 23, 35–42 doi: 10.1016/j.cois.2017.06.008 29129280

[91] Trowbridge A.M., Daly R.W., Helmig D., Stoy P.C. and Monson R.K. (2014), Herbivory and climate interact serially to control monoterpene emissions from pinyon pine forests. Ecology, 95: 1591–1603. doi: 10.1890/13-0989.1 25039223

[92] Stoy P. C., Trowbridge A.M., Siqueira M.B., Souza Freire L., Phillips R.P., Jacobs L., et al, 2021. Vapor pressure deficit helps explain biogenic volatile organic compound fluxes from the forest floor and canopy of a temperate deciduous forest. Oecologia 202, 1–18. doi: 10.1007/s00442-021-04891-1 33677772

[93] Sáenz-Romero C., Rehfeldt G.E., Crookston N.L., Duval P., Beaulieu J., 2012. Spline models of contemporary, 2030, 2060 and 2090 climates for Michoacan state, México. Impacts on the vegetation. Rev. Fitotecnia Mex. 35, 333–345. doi: 10.35196/rfm.2012.4.333

[94] Bentz B.J., Logan J.A., Amman G.D., 1991. Temperature-dependent development of the mountain pine beetle (Coleoptera: Scolytidae) and simulation of its phenology. Can. Entomol. 123, 1083–1094. doi: 10.4039/ent1231083-5

[95] Tykarski P., 2006. Beetles associated with scolytids (Coleoptera, Scolytidae) and the elevational gradient: Diversity and dynamics of the community in the Tatra National Park, Poland. Forest Ecol. Manage. 225,146–159. doi: 10.1016/j.foreco.2005.12.034

[96] Raffa K.F., Aukema B.H., Bentz B.J., Carroll A.L., Hicke J.A., Kolb T.E., 2015. Responses of Tree-killing Bark Beetles to a changing climate. In: Björkman C. and Niemelä P. Climante Change and Insect Pests. CAB International. p. 173–201. doi: 10.1079/9781780643786.0173

[97] Peñuelas J., Oyaga R., Boada M., Jump A.S., 2007. Migration, invasion and decline: changes in recruitment and forest structure in a warming-linked shift of European beech forest in Catalonia (NE Spain). Ecography 30, 830–838. doi: 10.1111/j.2007.0906-7590.05247.x

[98] Lenoir J. Gégout J.C., Marquet P.A, de Ruffray P., Brisse H., 2008. A significant upward shift in plant species optimum elevation during the 20th Century. Science 320, 1768–1770. doi: 10.1126/science.1156831 18583610

[99] Sáenz-Romero C., Lindig-Cisneros R.A., Joyce D.G., Beaulieu J., St-Clair J.B., Jaquish B.C., 2016. Assisted migration of forest populations for adapting trees to climate change (Migración asistida de las poblaciones forestales para la adaptación de árboles ante el cambio climático). Revista Chapingo Serie Ciencias Forestales y del Ambiente 22(3): 303–323. doi: 10.5154/r.rchscfa.2014.10.052

[100] Six D.L., 2012. Ecological and evolutionary determinants of barkbeetle-fungus symbioses. Insects 3, 339–366. doi: 10.3390/insects3010339 26467964 PMC4553632

[101] Six D.L., 2020. A major symbiont shift supports a major niche shift in a clade of tree-killing bark beetles. Ecol. Entomol., 45, 190–201. doi: 10.1111/een.12786

[102] Coulson R. N., Klepzig K. D. (Eds.), 2011. Southern Pine Beetle II. Southern Research Station. Gen. Tech. Rep. SRS-140 Ashville, NC. USA. 512 p.

[103] Mendoza-Villa O. N., Obregón Zúñiga A., 2016. Cambio en la abundancia de Dendroctonus frontalis Zimmerman, 1868 y Dendroctonus mexicanus Hopkins, 1909 (Coleóptera: Curculionidae: Scolytinae) en un gradiente altitudinal en el cerro “La Pingüica”, Pinal de Amoles, Querétaro. Entomol. Forest. Mex. 3, 644–648.

[104] Stadelmann G. et al. 2013. A predictive framework to assess spatio-temporal variability by the European spruce bark beetle.–Ecography 36: 1208–1217.

[105] Kulakowski et al 2017.

[106] Fettig Christopher J., Mortenson Leif A., Bulaon Beverly M., Foulk Patra B., 2019. Tree mortality following drought in the central and southern Sierra Nevada, California, U.S. Forest Ecology and Management, 432, 164–178, doi: 10.1016/j.foreco.2018.09.006

[107] Koontz M.J., Latimer A.M., Mortenson L.A. et al. Cross-scale interaction of host tree size and climatic water deficit governs bark beetle-induced tree mortality. Nat Commun 12, 129 (2021). doi: 10.1038/s41467-020-20455-y 33420082 PMC7794511

[108] Hayes C.J., DeGomez T.E., Clancy K.M., Williams K.K., McMilling J.D., Anhold J.A., 2008. Evaluation of funnel traps for characterizing the bark beetkle (Coleoptera: Scolytidae) communities in Ponderosa pine forest of North-Central Arizona. J. Econ. Entomol. 10,1253–1265. doi: 10.1093/jee/101.4.1253 18767735

[109] Guzmán-Aguilar G, Carbajal-Navarro A., Sáenz-Romero C., Herrerías-Diego Y., López-Toledo L., Blanco-García A., 2020. Abies religiosa seedling limitations for passive restoration practices at the Monarch Butterfly Biosphere Reserve in Mexico. Frontiers in Ecology and Evolution. 8(115):1–10. doi: 10.3389/fevo.2020.00115

