# Supplementary figures and images for: Abundance of Dendroctonus frontalis and D. mexicanus (Coleoptera: Scolytinae) along altitudinal transects in Mexico: Implications of climatic change for forest conservation
Source: PLoS One. 2023 Jul 5;18(7):e0288067. doi: 10.1371/journal.pone.0288067 (PMC10321627; doi:10.1371/journal.pone.0288067)

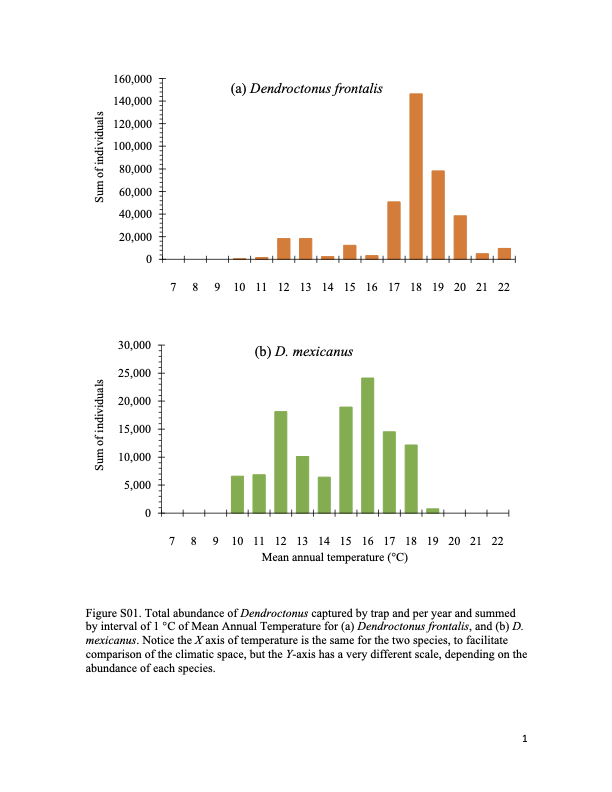

Supplement: S1 Fig — Notice the X axis of temperature is the same for the two species, to facilitate comparison of the climatic space, but the Y-axis has a very different scale, depending on the abundance of each species. (TIFF) [file pone.0288067.s001.tiff]

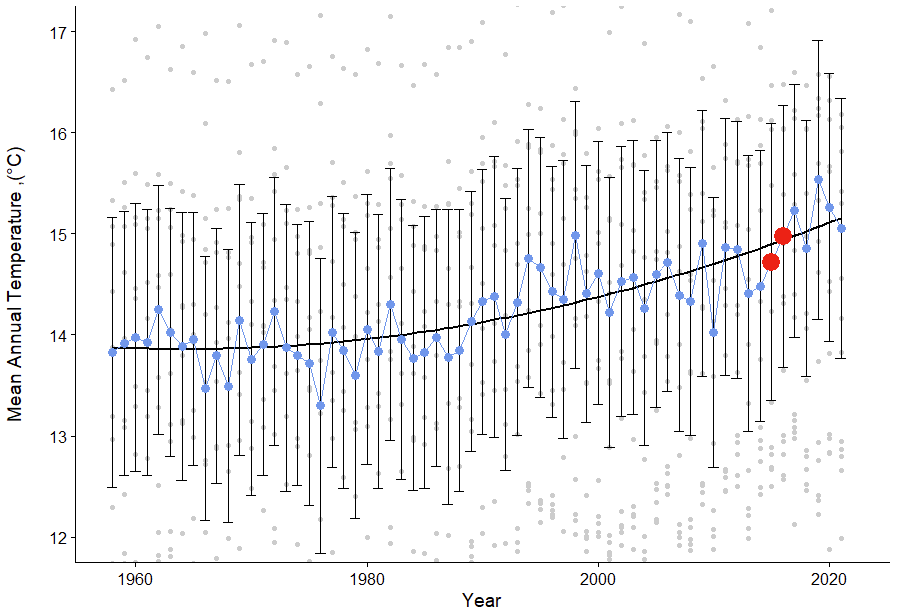

Supplement: S2 Fig — Average (across the center of all altitudinal transects) of MAT (light blue dots) is connected by a line to show annual variability (light blue line) and by a 10-year trendline (loess, span = 10, solid black line) to show the overall trend. Average across transects of the studied 2015–2016 years are highlighted as large red dots. Background gray dots are the MAT for the center of each transect (the coldest and warmest transects are omitted here due to Y-axis scale reasons, although their values were included to obtain the overall yearly averages). Vertical bars are 95% confidence interval in the all-transect annual MAT values. Data from TerraClimate website. (TIF) [file pone.0288067.s002.tif]
