# Supplementary material for: Abundance of Dendroctonus frontalis and D. mexicanus (Coleoptera: Scolytinae) along altitudinal transects in Mexico: Implications of climatic change for forest conservation
Source: PLoS One. 2023 Jul 5;18(7):e0288067. doi: 10.1371/journal.pone.0288067 (PMC10321627; doi:10.1371/journal.pone.0288067)
Supplement: S1 File — (DOCX) [file pone.0288067.s003.docx]

**Tables S01 to S11 (one Table per state), with details of transects (coordinates and altitude), sorted by state, from North to South and West to East.**

Table S01. Altitudinal transects on the state of Chihuahua, northwestern México.

| **State** | **Region** | **Tran.** | **Trap** | **Site** | **Lat N** | **Long W** | **Altitude (m)** |
| --- | --- | --- | --- | --- | --- | --- | --- |
| CHIH | Bocoyna | 1 | 3 | El Cuervito | 27.949 | -107.776 | 2458 |
| CHIH | Bocoyna | 1 | 2 | El Cuervito | 27.950 | -107.770 | 2444 |
| CHIH | Bocoyna | 1 | 4 | El Cuervito | 27.950 | -107.776 | 2400 |
| CHIH | Bocoyna | 1 | 1 | El Cuervito | 27.951 | -107.769 | 2362 |
| CHIH | Bocoyna | 2 | 2 | El Álamo | 28.038 | -107.600 | 2536 |
| CHIH | Bocoyna | 2 | 4 | El Álamo | 28.042 | -107.596 | 2519 |
| CHIH | Bocoyna | 2 | 1 | El Álamo | 28.036 | -107.602 | 2474 |
| CHIH | Bocoyna | 2 | 3 | El Álamo | 28.037 | -107.596 | 2466 |
| CHIH | Bocoyna | 3 | 4 | Los Basiguares | 27.990 | -107.549 | 2648 |
| CHIH | Bocoyna | 3 | 3 | Los Basiguares | 27.994 | -107.547 | 2583 |
| CHIH | Bocoyna | 3 | 2 | Los Basiguares | 28.002 | -107.547 | 2515 |
| CHIH | Bocoyna | 3 | 1 | Los Basiguares | 28.004 | -107.548 | 2442 |

CHICH = Chihuahua

Table S02. Altitudinal transects on the state of Coahuila, northeaster México.

| **State** | **Region** | **Tran.** | **Trap** | **Site** | **Lat N** | **Long W** | **Altitude (m)** |
| --- | --- | --- | --- | --- | --- | --- | --- |
| COAH | Arteaga | 1 | 8 | S. S. A. Alazanas | 25.274 | -100.464 | 3350 |
| COAH | Arteaga | 1 | 7 | S. S. A. Alazanas | 25.272 | -100.464 | 3250 |
| COAH | Arteaga | 1 | 6 | S. S. A. Alazanas | 25.270 | -100.464 | 3150 |
| COAH | Arteaga | 1 | 5 | S. S. A. Alazanas | 25.263 | -100.461 | 3050 |
| COAH | Arteaga | 1 | 4 | S. S. A. Alazanas | 25.289 | -100.509 | 2950 |
| COAH | Arteaga | 1 | 3 | S. S. A. Alazanas | 25.289 | -100.515 | 2850 |
| COAH | Arteaga | 1 | 2 | S. S. A. Alazanas | 25.290 | -100.519 | 2750 |
| COAH | Arteaga | 1 | 1 | S. S. A. Alazanas | 25.289 | -100.525 | 2650 |
| COAH | Arteaga | 2 | 8 | S. S. A. Alazanas | 25.289 | -100.491 | 3350 |
| COAH | Arteaga | 2 | 7 | S. S. A. Alazanas | 25.289 | -100.493 | 3250 |
| COAH | Arteaga | 2 | 6 | S. S. A. Alazanas | 25.289 | -100.495 | 3150 |
| COAH | Arteaga | 2 | 5 | S. S. A. Alazanas | 25.290 | -100.498 | 3050 |
| COAH | Arteaga | 2 | 4 | S. S. A. Alazanas | 25.292 | -100.504 | 2950 |
| COAH | Arteaga | 2 | 3 | S. S. A. Alazanas | 25.293 | -100.508 | 2850 |
| COAH | Arteaga | 2 | 2 | S. S. A. Alazanas | 25.293 | -100.514 | 2750 |
| COAH | Arteaga | 2 | 1 | S. S. A. Alazanas | 25.292 | -100.522 | 2650 |

COAH = Coahuila, S. S. A. Sierra San Antonio de las Alazanas

Table S03. Altitudinal transects on the state of Nuevo León, northeastern México.

| **State** | **Region** | **Tran.** | **Trap** | **Site** | **Lat N** | **Long W** | **Altitude (m)** |
| --- | --- | --- | --- | --- | --- | --- | --- |
| NL | Santiago | 1 | 8 | La Peñita | 25.340 | -100.368 | 2650 |
| NL | Santiago | 1 | 7 | La Peñita | 25.341 | -100.368 | 2550 |
| NL | Santiago | 1 | 6 | La Peñita | 25.343 | -100.368 | 2450 |
| NL | Santiago | 1 | 5 | La Peñita | 25.345 | -100.367 | 2350 |
| NL | Santiago | 1 | 4 | La Peñita | 25.347 | -100.366 | 2250 |
| NL | Santiago | 1 | 3 | La Peñita | 25.350 | -100.364 | 2150 |
| NL | Santiago | 1 | 2 | La Peñita | 25.352 | -100.362 | 2050 |
| NL | Santiago | 1 | 1 | La Peñita | 25.358 | -100.370 | 1950 |
| NL | Santiago | 2 | 8 | La Peñita (S. Seb.) | 25.338 | -100.374 | 2650 |
| NL | Santiago | 2 | 7 | La Peñita (S. Seb.) | 25.339 | -100.374 | 2550 |
| NL | Santiago | 2 | 6 | La Peñita (S. Seb.) | 25.341 | -100.374 | 2450 |
| NL | Santiago | 2 | 5 | La Peñita (S. Seb.) | 25.344 | -100.375 | 2350 |
| NL | Santiago | 2 | 4 | La Peñita (S. Seb.) | 25.346 | -100.375 | 2250 |
| NL | Santiago | 2 | 3 | La Peñita (S. Seb.) | 25.350 | -100.375 | 2150 |
| NL | Santiago | 2 | 2 | La Peñita (S. Seb.) | 25.355 | -100.376 | 2050 |
| NL | Santiago | 2 | 1 | La Peñita (S. Seb.) | 25.361 | -100.377 | 1950 |

NL = Nuevo León, S. Seb. = San Sebastián

Table S04. Altitudinal transects on the state of Durango, northwestern Mexico.

| **State** | **Region** | **Tran.** | **Trap** | **Site** | **Lat N** | **Long W** | **Altitude (m)** |
| --- | --- | --- | --- | --- | --- | --- | --- |
| DGO | San Dimas | 1 | 4 | El Lucero | 24.321 | -105.582 | 2600 |
| DGO | San Dimas | 1 | 3 | Río Miravalles | 24.287 | -105.548 | 2500 |
| DGO | San Dimas | 1 | 2 | Río Miravalles | 24.291 | -105.550 | 2400 |
| DGO | San Dimas | 1 | 1 | Río Miravalles | 24.303 | -105.551 | 2300 |
| DGO | San Dimas | 2 | 4 | El Lucero | 24.316 | -105.580 | 2600 |
| DGO | San Dimas | 2 | 3 | Río Miravalles | 24.283 | -105.550 | 2500 |
| DGO | San Dimas | 2 | 2 | Río Miravalles | 24.288 | -105.553 | 2400 |
| DGO | San Dimas | 2 | 1 | Río Miravalles | 24.305 | -105.556 | 2300 |

DGO = Durango

Table S05. Altitudinal transects on the state of Jalisco, western México.

| **State** | **Region** | **Tran.** | **Trap** | **Site** | **Lat N** | **Long W** | **Altitude (m)** |
| --- | --- | --- | --- | --- | --- | --- | --- |
| JAL | Tecolotlán | 1 | 3 | Sierra de Quila | 20.306 | -104.017 | 2306 |
| JAL | Tecolotlán | 1 | 4 | Sierra de Quila | 20.317 | -104.032 | 2196 |
| JAL | Tecolotlán | 1 | 2 | Sierra de Quila | 20.316 | -104.060 | 2096 |
| JAL | Tecolotlán | 1 | 1 | Sierra de Quila | 20.317 | -104.071 | 1999 |
| JAL | Tecolotlán | 2 | 3 | Sierra de Quila | 20.297 | -104.017 | 2299 |
| JAL | Tecolotlán | 2 | 4 | Sierra de Quila | 20.302 | -104.031 | 2199 |
| JAL | Tecolotlán | 2 | 2 | Sierra de Quila | 20.303 | -104.061 | 2099 |
| JAL | Tecolotlán | 2 | 1 | Sierra de Quila | 20.304 | -104.079 | 2003 |

JAL = Jalisco

Table S06. Altitudinal transects on the state of Querétaro, central México.

| **State** | **Region** | **Tran.** | **Trap** | **Site** | **Lat N** | **Long W** | **Altitude (m)** |
| --- | --- | --- | --- | --- | --- | --- | --- |
| QRO | Arroyo Seco | 1 | 5 | El Tepozán | 21.361 | -99.773 | 2003 |
| QRO | Arroyo Seco | 1 | 4 | El Tepozán | 21.362 | -99.771 | 1931 |
| QRO | Arroyo Seco | 1 | 3 | El Tepozán | 21.364 | -99.766 | 1827 |
| QRO | Arroyo Seco | 1 | 2 | El Tepozán | 21.361 | -99.747 | 1733 |
| QRO | Arroyo Seco | 1 | 1 | El Tepozán | 21.377 | -99.771 | 1587 |
| QRO | Landa Matamoros | 2 | 4 | El Madroño | 21.282 | -99.149 | 1716 |
| QRO | Landa Matamoros | 2 | 2 | El lobo | 21.298 | -99.123 | 1670 |
| QRO | Landa Matamoros | 2 | 3 | El Madroño | 21.281 | -99.149 | 1651 |
| QRO | Landa Matamoros | 2 | 1 | El lobo | 21.298 | -99.124 | 1586 |
| QRO | Pinal de Amoles | 3 | 8 | La Pingüica | 21.162 | -99.693 | 3058 |
| QRO | Pinal de Amoles | 3 | 7 | La Pingüica | 21.125 | -99.682 | 2904 |
| QRO | Pinal de Amoles | 3 | 6 | La Pingüica | 21.125 | -99.678 | 2790 |
| QRO | Pinal de Amoles | 3 | 5 | La Pingüica | 21.126 | -99.675 | 2706 |
| QRO | Pinal de Amoles | 3 | 4 | La Pingüica | 21.122 | -99.675 | 2610 |
| QRO | Pinal de Amoles | 3 | 3 | La Pingüica | 21.128 | -99.664 | 2541 |
| QRO | Pinal de Amoles | 3 | 2 | La Pingüica | 21.130 | -99.633 | 2468 |
| QRO | Pinal de Amoles | 3 | 1 | La Pingüica | 21.132 | -99.627 | 2393 |

QRO = Querétaro

Table S07. Altitudinal transects on the state of Hidalgo, central México.

| **State** | **Region** | **Tran.** | **Trap** | **Site** | **Lat N** | **Long W** | **Altitude (m)** |
| --- | --- | --- | --- | --- | --- | --- | --- |
| HGO | Pacula | 1 | 8 | Los Mármoles | 20.921 | -99.233 | 2117 |
| HGO | Zimapán | 1 | 1 | Los Mármoles | 20.931 | -99.227 | 2098 |
| HGO | Zimapán | 1 | 2 | Los Mármoles | 20.934 | -99.225 | 1998 |
| HGO | Pacula | 1 | 7 | Los Mármoles | 20.945 | -99.232 | 1880 |
| HGO | Zimapán | 1 | 3 | Los Mármoles | 20.937 | -99.222 | 1880 |
| HGO | Zimapán | 1 | 4 | Los Mármoles | 20.937 | -99.221 | 1812 |
| HGO | Zimapán | 1 | 5 | Los Mármoles | 20.938 | -99.219 | 1722 |
| HGO | Zimapán | 1 | 6 | Los Mármoles | 20.938 | -99.218 | 1654 |
| HGO | Zimapán | 2 | 4 | Los Mármoles | 20.938 | -99.242 | 2120 |
| HGO | Zimapán | 2 | 3 | Los Mármoles | 20.940 | -99.244 | 2024 |
| HGO | Zimapán | 2 | 2 | Los Mármoles | 20.946 | -99.245 | 1938 |
| HGO | Zimapán | 2 | 1 | Los Mármoles | 20.948 | -99.246 | 1881 |
| HGO | Jacala Ledezma | 3 | 4 | San Nicolas | 20.901 | -99.156 | 2611 |
| HGO | Jacala Ledezma | 3 | 3 | San Nicolas | 20.904 | -99.153 | 1532 |
| HGO | Jacala Ledezma | 3 | 2 | San Nicolas | 20.906 | -99.153 | 1440 |
| HGO | Jacala Ledezma | 3 | 1 | San Nicolas | 20.907 | -99.154 | 1342 |

HGO = Hidalgo

Table S08. Altitudinal transects on the state of Michoacán, western México.

| **State** | **Region** | **Tran.** | **Trap** | **Site** | **Lat N** | **Long W** | **Altitude (m)** |
| --- | --- | --- | --- | --- | --- | --- | --- |
| MICH | NSJP | 1 | 1 | Cerro de Pario | 19.471 | -102.182 | 2830 |
| MICH | NSJP | 1 | 2 | Cerro de Pario | 19.471 | -102.178 | 2730 |
| MICH | NSJP | 1 | 3 | Aguililla/Werocutin | 19.463 | -102.185 | 2630 |
| MICH | NSJP | 1 | 4 | La Alberca | 19.457 | -102.191 | 2530 |
| MICH | NSJP | 1 | 5 | Cuch Áporo | 19.449 | -102.185 | 2430 |
| MICH | NSJP | 1 | 6 | Salomé | 19.447 | -102.175 | 2330 |
| MICH | NSJP | 1 | 7 | Pascuala | 19.444 | -102.166 | 2230 |
| MICH | NSJP | 1 | 8 | Pinalosa | 19.444 | -102.156 | 2130 |
| MICH | NSJP | 2 | 1 | Cerro Prieto | 19.443 | -102.222 | 2889 |
| MICH | NSJP | 2 | 2 | El Derrumbadero | 19.447 | -102.221 | 2789 |
| MICH | NSJP | 2 | 3 | Juritzícuaro | 19.452 | -102.216 | 2689 |
| MICH | NSJP | 2 | 4 | La Alberca | 19.460 | -102.201 | 2589 |
| MICH | NSJP | 2 | 5 | El Destiladero | 19.458 | -102.196 | 2489 |
| MICH | NSJP | 2 | 6 | Copícuaro | 19.439 | -102.192 | 2389 |
| MICH | NSJP | 2 | 7 | El Maguey | 19.426 | -102.183 | 2289 |
| MICH | NSJP | 2 | 8 | Rosario | 19.429 | -102.170 | 2189 |

MICH = Michoacán, NSJP = Nuevo San Juan Parangaricutiro

Table S09. Altitudinal transects on the state of México, central México.

| **State** | **Region** | **Tran.** | **Trap** | **Site** | **Lat N** | **Long W** | **Altitude (m)** |
| --- | --- | --- | --- | --- | --- | --- | --- |
| EDO MEX | Tlalmanalco | 1 | 8 | Aculco | 19.250 | -98.669 | 3611 |
| EDO MEX | Tlalmanalco | 1 | 7 | Aculco | 19.258 | -98.671 | 3551 |
| EDO MEX | Tlalmanalco | 1 | 6 | Aculco | 19.267 | -98.668 | 3463 |
| EDO MEX | Tlalmanalco | 1 | 5 | Aculco | 19.272 | -98.670 | 3410 |
| EDO MEX | Tlalmanalco | 1 | 4 | Aculco | 19.280 | -98.670 | 3323 |
| EDO MEX | Tlalmanalco | 1 | 3 | Aculco | 19.283 | -98.673 | 3269 |
| EDO MEX | Tlalmanalco | 1 | 2 | Aculco | 19.301 | -98.671 | 3220 |
| EDO MEX | Tlalmanalco | 1 | 1 | Aculco | 19.300 | -98.660 | 3152 |

EDO MEX = Estado de México

Table S10. Altitudinal transects on the state of Oaxaca, southern México.

| **State** | **Region** | **Tran.** | **Trap** | **Site** | **Lat N** | **Long W** | **Altitude (m)** |
| --- | --- | --- | --- | --- | --- | --- | --- |
| OAX | S. C. Lachatao | 1 | 8 | Pblo. Man. | 17.202 | -96.484 | 2869 |
| OAX | S. C. Lachatao | 1 | 7 | Pblo. Man. | 17.200 | -96.485 | 2758 |
| OAX | S. C. Lachatao | 1 | 6 | Pblo. Man. | 17.197 | -96.487 | 2660 |
| OAX | S. C. Lachatao | 1 | 5 | Pblo. Man. | 17.193 | -96.489 | 2555 |
| OAX | S. C. Lachatao | 1 | 4 | Pblo. Man. | 17.192 | -96.494 | 2459 |
| OAX | S. C. Lachatao | 1 | 3 | Pblo. Man. | 17.191 | -96.497 | 2360 |
| OAX | S. C. Lachatao | 1 | 2 | Pblo. Man. | 17.186 | -96.499 | 2255 |
| OAX | S. C. Lachatao | 1 | 1 | Pblo. Man. | 17.203 | -96.509 | 2161 |
| OAX | S. R. Pápalo | 2 | 8 | S. R. Pápalo | 17.775 | -96.838 | 2899 |
| OAX | S. R. Pápalo | 2 | 7 | S. R. Pápalo | 17.774 | -96.841 | 2799 |
| OAX | S. R. Pápalo | 2 | 6 | S. R. Pápalo | 17.772 | -96.844 | 2658 |
| OAX | S. R. Pápalo | 2 | 5 | S. R. Pápalo | 17.770 | -96.852 | 2569 |
| OAX | S. R. Pápalo | 2 | 4 | S. R. Pápalo | 17.768 | -96.857 | 2456 |
| OAX | S. R. Pápalo | 2 | 3 | S. R. Pápalo | 17.769 | -96.863 | 2345 |
| OAX | S. R. Pápalo | 2 | 2 | S. R. Pápalo | 17.771 | -96.865 | 2242 |
| OAX | S. R. Pápalo | 2 | 1 | S. R. Pápalo | 17.774 | -96.866 | 2113 |

OAX = Oaxaca, S. C. = Santa Catarina, S. R. = Santos Reyes, Pblo. Man. = Pueblos Mancomunados.

Table S11. Altitudinal transects on the state of Chiapas, southeastern México.

| State | Region | Tran. | Trap | Site | Lat N | Long W | Altitude (**m**) |
| --- | --- | --- | --- | --- | --- | --- | --- |
| CHIS | Motozintla | 1 | 5 | Plan Grande | 15.339 | -92.251 | 2134 |
| CHIS | Motozintla | 1 | 2 | Rivera Morelos | 15.333 | -92.251 | 2037 |
| CHIS | Motozintla | 1 | 6 | Plan Grande | 15.345 | -92.228 | 2034 |
| CHIS | Motozintla | 1 | 1 | Rivera Morelos | 15.331 | -92.251 | 2033 |
| CHIS | Motozintla | 1 | 3 | Rivera Morelos | 15.335 | -92.251 | 1929 |
| CHIS | Motozintla | 1 | 7 | Plan Grande | 15.345 | -92.228 | 1899 |
| CHIS | Motozintla | 1 | 8 | Plan Grande | 15.346 | -92.226 | 1838 |
| CHIS | Motozintla | 1 | 10 | El Dormido | 15.359 | -92.229 | 1775 |
| CHIS | Motozintla | 1 | 4 | El Dormido | 15.336 | -92.252 | 1757 |
| CHIS | Motozintla | 1 | 9 | Plan Grande | 15.356 | -92.230 | 1743 |
| CHIS | Las Margaritas | 2 | 1 | Ojo de Agua II | 16.262 | -91.809 | 1522 |
| CHIS | Las Margaritas | 2 | 2 | Ojo de Agua II | 16.263 | -91.796 | 1505 |
| CHIS | Las Margaritas | 2 | 3 | San Calampio | 16.281 | -91.794 | 1457 |
| CHIS | Las Margaritas | 2 | 4 | San Calampio | 16.287 | -91.787 | 1437 |

CHIS = Chiapas.
